# A keratinocyte-adipocyte signaling loop is reprogrammed by loss of BTG3 to augment skin carcinogenesis

**DOI:** 10.1038/s41418-024-01304-7

**Published:** 2024-05-07

**Authors:** Yu-Che Cheng, Jack Dalit Acedera, Yi-Ju Li, Sheau-Yann Shieh

**Affiliations:** 1 Institute of Biomedical Sciences, Academia Sinica, Taipei, Taiwan; 2 Taiwan International Graduate Program in Molecular Medicine, National Yang Ming Chiao Tung University and Academia Sinica, Taipei, Taiwan

**Keywords:** Tumour-suppressor proteins, Cancer microenvironment

## Abstract

Obesity is endemic to many developed countries. Overweight or obesity is associated with an increased risk of developing cancer. Dysfunctional adipose tissue alters cancer cell proliferation and migration; however, whether and how neoplastic epithelial cells communicate with adipose tissue and the underlying mechanism are less clear. BTG3 is a member of the anti-proliferative BTG/Tob family and functions as a tumor suppressor. Here, we demonstrated that BTG3 levels are downregulated in basal cell carcinoma and squamous cell carcinoma compared to normal skin tissue, and *Btg3* knockout in mice augmented the development of papilloma in a mouse model of DMBA/TPA-induced skin carcinogenesis. Mechanistically, BTG3-knockout keratinocytes promoted adipocyte differentiation mainly through the release of IL1α, IL10, and CCL4, as a result of elevated NF-κB activity. These adipocytes produced CCL20 and FGF7 in a feedback loop to promote keratinocyte migration. Thus, our findings showcased the role of BTG3 in guarding the interplay between keratinocytes and adjacent adipocytes, and identified the underlying neoplastic molecular mediators that may serve as possible targets in the treatment of skin cancer.

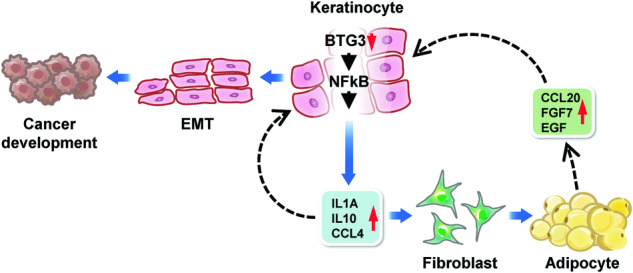

## Introduction

Skin cancers, including cutaneous melanoma and non-melanoma skin cancer (NMSC), arise from genetic predisposition and exposure to environmental risk factors, such as sunlight^1^. Across the disease spectrum, NMSC represent approximately 95% of skin cancers and can be further divided into two main subtypes: cutaneous squamous cell carcinoma (SCC) and basal cell carcinoma (BCC) [2–4]. Several studies have suggested that the global incidence of NMSC has increased by 3–8% annually since 1960, and it is 18–20 times higher than the incidence of melanoma [5, 6]. Owing to the increasing prevalence of skin cancers and the challenges in efficient treatment, it is increasingly important to identify possible ways to prevent or cure the disease.

The skin is an organ that protects the body from harmful external intrusions, such as UV radiation and chemical and physical damage. Human skin is composed of the epidermis, dermis, and subcutaneous adipose tissue. The epidermis is the outermost layer of the skin and can be subdivided into the stratum corneum, lucidum, granulosum, spinosum, and the basal layer. Basal keratinocytes replenish the epidermis through proliferation and differentiation. Keratinocyte dysfunction has been linked to several skin diseases, including cancer^7,8^. The dermis, which is the inner layer of the skin, contains connective tissue, hair follicles, blood vessels, lymphatic vessels, and sweat glands. The deeper subcutaneous layer is composed of adipose and connective tissue [9, 10].

Adipocytes are the major constituents of adipose tissue and they play key roles in the homeostatic control of body metabolism [11]. Upon caloric excess, adipocytes become hypertrophic, leading to various physiological stresses, such as hypoxia, oxidative stress, and disruptions in the protein secretory pathway. Under these conditions, adipocytes become dysfunctional and less efficient at fulfilling their original roles in the storage and neutralization of potentially harmful lipids. Adipocyte dysfunction has been linked to obesity-related metabolic disorders, such as insulin resistance, type 2 diabetes, cardiovascular disease, chronic inflammation, and cancer [12–14]. Dysfunctional adipocytes release mitogenic and inflammatory factors that affect surrounding cells, such as fibroblasts, immune cells, and cancer cells. For example, adipocyte-released leptin promotes epithelial–mesenchymal transition (EMT) in breast cancer cells by activating the PI3K/AKT signaling pathway, and TNF-α and IL-6 secretion upregulates PD-L1 in cancer cells to promote immune evasion [15, 16]. Although various downstream effects have been observed, the molecular basis of adipocyte dysfunction and the involvement of tumor-associated genes are still not fully understood.

*BTG3* is a member of the antiproliferative *BTG* (B-cell translocation gene)/*Tob* (Transducer of ErbB2) gene family, which also includes *BTG1*, *BTG2*, *BTG4*, *TOB*, and *TOB2*. Members of the BTG family have very similar N-terminal domains, but appear to diverge at their C-termini [17]. Btg3-deficient (*Btg3*^–/–^) mice display a higher incidence of lung tumors, and the downregulation of BTG3 has been observed in renal, breast, and prostate cancers [18–20], suggesting that BTG3 may play a role in suppressing tumorigenesis. In our previous studies, we demonstrated that BTG3 interacts with and suppresses AKT, a kinase that is frequently dysregulated in cancer. Upon overexpression, BTG3 suppresses tumorigenesis and EMT through the AKT−GSK3β−β-catenin signaling pathway [21]. Furthermore, BTG3 inhibits HIF1 activity by competing with the co-activator p300 for HIF1α interaction, and suppresses HIF1-mediated angiogenesis [22].

Here, we describe a unique paracrine-mediated interaction between BTG3-deleted keratinocytes and adipocytes, which may account for the increased incidence of skin carcinogenesis caused by BTG3 downregulation.

## Results

### Medium conditioned by BTG3-knockout keratinocytes promotes 3T3-L1 adipocyte differentiation

**Fig. 1:**
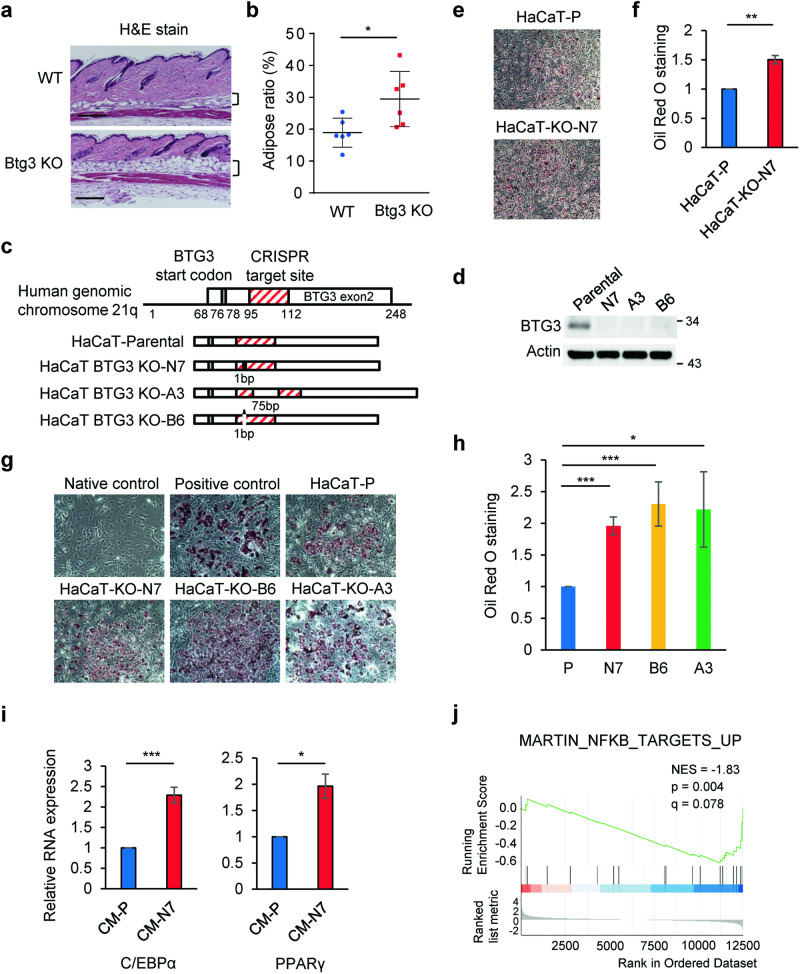
BTG3-knockout keratinocytes promote 3T3-L1 adipogenic differentiation. **a, b** Hematoxylin and eosin staining of the back skin from 8-week old wild-type (WT) and *Btg3-*knockout (KO) mice. The hypodermal adipose layer of the back skin was thicker in *Btg3*^−/−^ mice than WT mice. Quantified results are shown in **b**. Brackets indicate the adipose layer. **c** Schematic diagrams of the CRISPR gRNA target site in the *BTG3* gene and the resulting changes in the three HaCaT BTG3-KO clones, N7, A3, and B6. **d** Expression levels of BTG3 in parental and BTG3-KO HaCaT cells, as revealed by western blotting. **e, f** Co-culture of BTG3-KO HaCaT with 3T3-L1 promoted 3T3-L1 adipocyte differentiation. Adipocytes were stained with Oil Red O **e** and quantified in **f**. **g, h** Enhanced 3T3-L1 adipocyte differentiation induced by conditioned media (CM) from BTG3-KO cells. 3T3-L1 differentiation was induced by CM from BTG3-KO and parental HaCaT cells and adipogenesis was determined by Oil Red O staining **g** and quantified in **h**. **i** Expression of adipogenesis-related genes C/EBPα and PPARγ was upregulated in BTG3-KO CM-differentiated adipocytes. Shown are results of RT-qPCR. **j** Gene set enrichment analysis comparing RNA-seq data from BTG3-KO and parental HaCaT cells. Expression of genes related to NF-κB pathway was enriched in BTG3-KO cells.

To identify potential impairments in *Btg3*-knockout (KO) mice, we performed whole-body dissection. Among the dissected tissues, the subcutaneous adipose tissue was noticeably thicker in KO mice, although the mice were not visually obese. Indeed, closer examination of 8-week old wild-type (WT) and *Btg3*^−/−^ mice revealed an increased ratio of adipose layer in the back skin in *Btg3*^−/−^ mice, suggesting that Btg3 may regulate adipose tissue formation (Fig. 1a and 1b).

There have been reports implicating the functional interplay between epidermal keratinocytes and hypodermal adipocytes in the hair cycle, wound healing, and scarring [23–25]. To explore the possible involvement of BTG3 in this interplay in the skin, we first performed in vitro studies using human keratinocyte HaCaT cells and the pre-adipocyte cell line, 3T3-L1. To understand the role of BTG3, three BTG3-KO HaCaT clones, N7, B6, and A3, were generated using the CRISPR/Cas9 approach (Fig. 1c), and ablation of BTG3 in these cells was confirmed by western blotting (Fig. 1d) and genome sequencing (Fig. S1a). Compared to the parental HaCaT cells, co-culture of BTG3-KO HaCaT with 3T3-L1 enhanced adipogenesis of the latter (Fig. 1e and 1f). To dissect this effect further, conditioned media (CM) were collected from parental and BTG3-KO HaCaT cells and compared for their ability to induce adipocyte differentiation in 3T3-L1 cells. The results showed that BTG3-KO CM promoted 3T3-L1 cell differentiation, as evidenced by the increased number of oil droplets in the cells (Fig. 1g and 1h). In agreement, the RNA expression of adipogenesis-related genes such as C/EBPα and PPARγ (Fig. 1i) and the protein expression levels of C/EBPα (Fig. S2) were higher in adipocytes differentiated with BTG3-KO CM than those with parental CM, assessed by reverse transcription quantitative polymerase chain reaction (RT-qPCR) and immunocytochemistry, respectively. To understand the underlying changes caused by BTG3 deletion, RNA sequencing (RNA-seq) was employed to compare transcriptomic profiles of parental and BTG3-KO HaCaT (Fig. S3a). Interestingly, gene set enrichment analysis (GSEA) indicated a possible functional link between BTG3 loss and the NF-κB pathways^26^ (Fig. 1j). Taken together, these data suggest that BTG3 deletion in keratinocytes promotes adipocyte differentiation via paracrine secretion.

### BTG3-KO keratinocytes promote 3T3-L1 adipogenesis through the release of IL1α, IL10, and CCL4

**Fig. 2:**
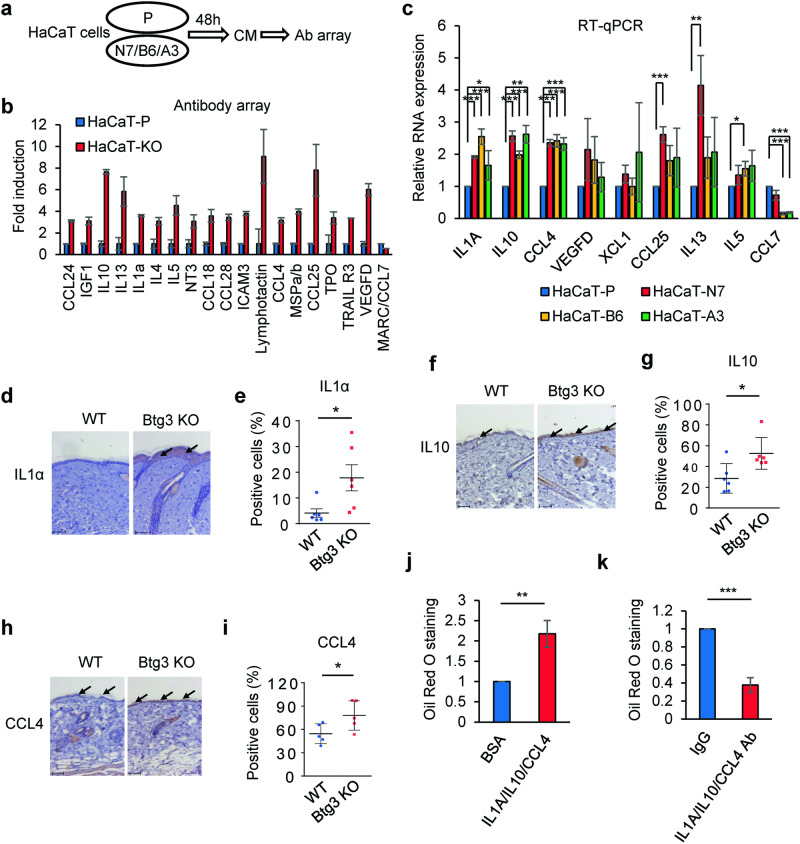
BTG3-KO keratinocytes release IL1α, IL10, and CCL4 to promote 3T3-L1 adipogenic differentiation. **a** Schematic workflow of cytokine antibody array analysis. CM was collected after 48 h of culture, and analyzed using a human cytokine antibody array. BTG3-KO CM was a mixture from the three KO clones indicated. **b** The levels of factors and cytokines, including IL1α, IL10, and CCL4, were increased in BTG3-KO CM. CM from (a) was subjected to antibody array analysis. Signals from duplicated spots were quantified and compared between parental and BTG3-KO cells. CCL7 is shown as an unaltered control. **c** mRNA expression levels of *IL1A*, *IL10*, *CCL4*, and *VEGFD*, but not *CCL7*, were increased in BTG3-KO HaCaT cells. Reverse transcription quantitative polymerase chain reaction (RT-qPCR) was performed and results from three independent experiments are shown. **d–i** Elevated expression levels of IL1α, IL10, and CCL4 in the back skin of *Btg3*-KO mice. 8-week-old WT and *Btg3*-KO mice were shaved and their back skin was collected 3 weeks later and embedded for immunohistochemical (IHC) analysis using antibodies against IL1α **d**, IL10 **f**, and CCL4 **h**. Examples of positively stained cell are indicated by arrows. Quantified results (*n* = 6) are shown in **e**, **g**, and **i**, respectively. **j** Addition of recombinant IL1α, IL10, and CCL4 to parental CM promotes 3T3-L1 differentiation. Adipogenesis was measured by Oil Red O staining followed by quantification. **k** Antibody-mediated neutralization of IL1α, IL10 and CCL4 in BTG3-KO CM reduced the effect on 3T3-L1 adipogenic differentiation. *P < 0.05. **P < 0.01.

Next, we employed human cytokine antibody arrays to determine which secreted factors were involved in BTG3-KO HaCaT-cell-promoted adipogenesis. The results showed that the levels of several factors, including IL1α, IL10, CCL4, and VEGFD, were increased in the medium conditioned by BTG3-KO HaCaT cells when compared to the parental cells (Fig. 2a and 2b). The increase in mRNA expression levels of *IL1A*, *IL10*, *CCL4*, and *VEGFD* was confirmed by RT-qPCR in the three BTG3-KO lines (Fig. 2c). Additionally, we compared the expression levels of IL1α, IL10, and CCL4 in the back skin of WT and *Btg3*^−/−^ mice using immunohistochemistry (IHC). In agreement with the results in HaCaT cells, the levels of IL1α, IL10, and CCL4 were increased in *Btg3*^−/−^ mice (Fig. 2d–i).

To determine if the above-identified factors contributed to adipogenesis, we supplemented the CM collected from parental HaCaT cells with recombinant human IL1α, IL10, and CCL4 and used it in 3T3-L1 differentiation assays. The results showed that, compared to CM supplemented with bovine serum albumin (BSA), the IL1α-, IL10-, and CCL4-supplemented CM promoted 3T3-L1 adipogenesis (Fig. 2j). In contrast, neutralization of IL1α, IL10, and CCL4 in BTG3-KO CM using specific antibodies reduced the adipogenic differentiation of 3T3-L1 cells (Fig. 2k). Collectively, these results indicate that BTG3-KO keratinocytes promote adipogenesis through IL1α, IL10, and CCL4 secretion.

### BTG3 knockout enhances NF-κB activity in keratinocytes

**Fig. 3:**
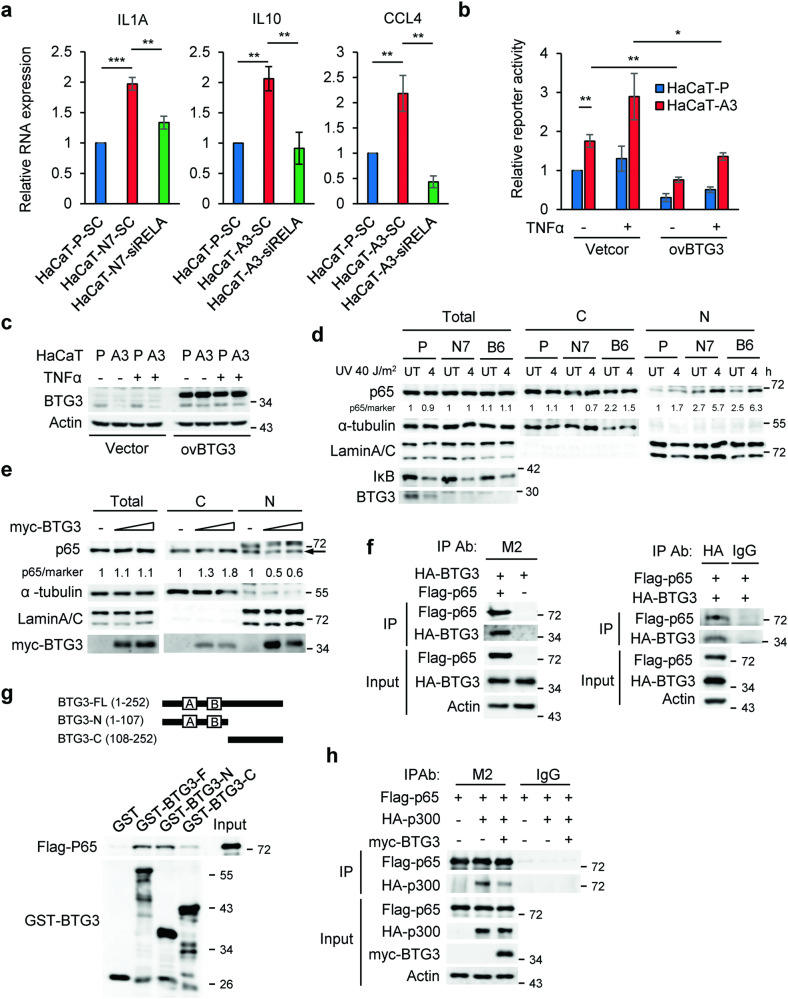
NFκB mediates the effect of BTG3 ablation on adipocyte differentiation. **a** Increased expression of IL1A, IL10, and CCL4 in BTG3-KO HaCaT cells required NFκB. RT-qPCR was performed with RNA prepared from control or *RELA*-siRNA-transfected parental and BTG3-KO HaCaT cells. Expression levels of *IL1A*, *IL10*, and *CCL4* were reduced as a result of RELA knockdown. **b, c** NFκB activity was inhibited by ectopic expression of BTG3. NF-κB luciferase reporter assays were conducted with or without overexpression of myc-BTG3 in the presence or absence of TNFα in parental or BTG3 KO HaCaT cells by transient transfection. *P < 0.05. **P < 0.01. Overexpression of BTG3 was assessed by western blotting **c**. **d** BTG3 KO increased nuclear distribution of p65 after UV treatment. Cytosolic and nuclear fractionation was performed with UV-irradiated parental and BTG3-KO (N7 and B6) HaCaT cells. The distribution of p65 was assessed by immunoblotting. Lamin A/C and α-tubulin were used as the nuclear and cytosolic markers, respectively. Numbers indicate relative levels of p65 after normalization to either α-tubulin or Lamin A/C**. e** Re**-**expression of BTG3 in BTG3 KO HaCaT cells decreased p65 nuclear distribution. **f, g** BTG3 interacted with p65 in vivo as demonstrated by coimmunoprecipitation **f** and in vitro in a GST pulldown assay with recombinant GST-BTG3 proteins **g**. 293 T cells were transfected with the indicated constructs and immunoprecipitation was conducted with either anti-Flag (M2) or anti-HA antibody. **h** BTG3 competed with p300 for binding p65 in 293 T cells as revealed by coimmunoprecipitation assays.

As IL1α, IL10, and CCL4 are direct targets of NF-κB [27–29], we therefore depleted RELA, a subunit of NF-κB, by siRNA in parental and BTG3-KO HaCaT cells, and assessed mRNA expression levels by RT-qPCR. The results demonstrated that knockdown of RELA abolished the increase of *IL1A*, *IL10*, and *CCL4* mRNA levels in BTG3-KO HaCaT cells (Fig. 3a). The possible inhibition of NF-κB by BTG3 was further confirmed by a reporter assay, in which overexpressed BTG3 downregulated the activity of a NF-κB luciferase reporter in HaCaT cells (Fig. 3b and 3c).

It has been reported that UV induces NF-κB phosphorylation, accumulation, nuclear localization, and activation [30]. To understand the mechanism underlying the regulation of NF-κB by BTG3, fractionation assays were performed to compare the cellular localization of NF-κB in parental and BTG3-KO HaCaT cells after UV. The results indicated that nuclear p65 levels increased more in BTG3-KO cells than in parental cells after UV radiation (Fig. 3d). In contrast, reexpression of BTG3 in BTG3-KO cells reduced nuclear p65 (Fig. 3e). Moreover, ectopically expressed p65 interacted with BTG3 in cells as demonstrated by coimmunoprecipitation (Fig. 3f). Physically, BTG3 interacts with p65 via its N-terminal domain in vitro (Fig. 3g). These data together suggest that BTG3 may inhibit p65 by preventing its nuclear localization. In agreement, we also observed decreased p65 interaction with its coactivator p300 upon coexpression of BTG3 (Fig. 3h). Taken together, these data demonstrate that BTG3 may act as a negative regulator of the NF-κB signaling pathway.

### BTG3-KO CM reprograms adipocytes to promote keratinocyte migration through secretion of the adipokines, CCL20 and FGF7

**Fig. 4:**
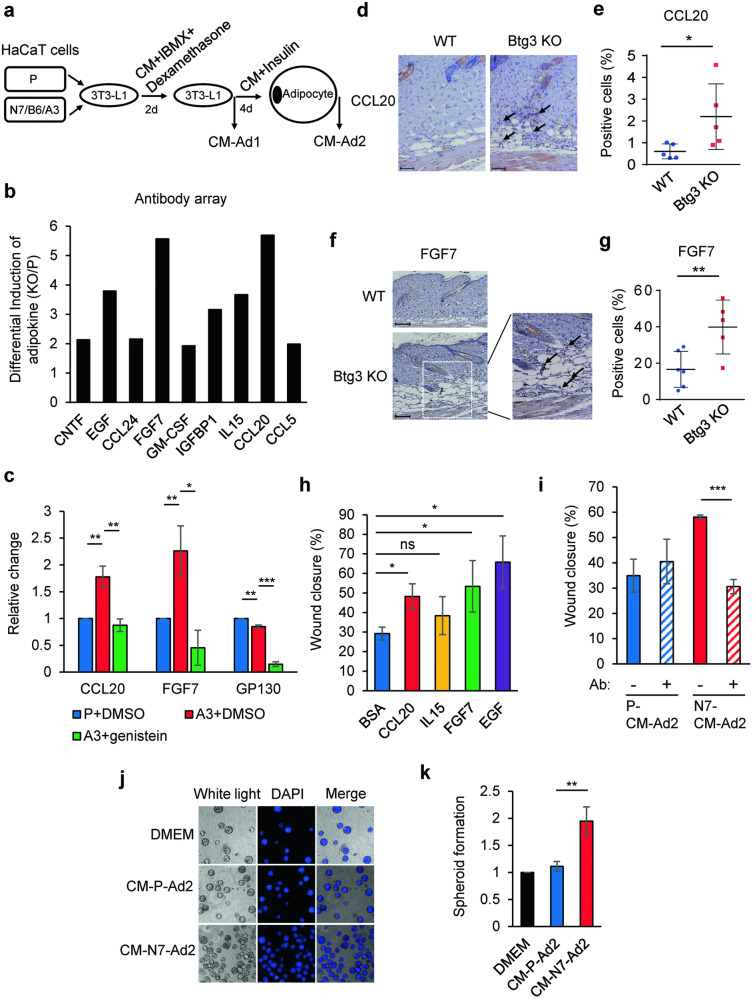
Adipokines CCL20 and FGF7 induced by BTG3-KO CM promote keratinocyte migration. **a** Workflow for collection of adipokines for antibody array analysis. CM collected at the start of differentiation (Ad1) and 4 d after differentiation (Ad2) were analyzed using an antibody array and compared. **b** Increased adipokine release by adipocytes differentiated in BTG3-KO CM. Factors and cytokines differentially expressed during the 4-d differentiation period were compared between BTG3-KO and parental CM. The ratio is shown. Release of CCL20, FGF7, EGF, and IL15 was higher from BTG3-KO-CM-induced adipocytes. **c** The increase in CCL20 and FGF7 was abolished by the adipocyte differentiation inhibitor, genistein, as measured by ELISA. GP130 was used as a positive control for genistein response. **d–g** Skin expression levels of CCL20 and FGF7 were elevated in *Btg3*^−/−^ mice compared to WT mice. Eight-week-old WT and *Btg3*-KO mice were shaved and sacrificed 3 weeks later. The expression levels of CCL20 and FGF7 in back skin tissue were determined by IHC staining with anti-CCL20 **d** or anti-FGF7 **f** antibodies. Arrows indicate positively stained cells. Quantified results are shown in **e** for CCL20 (*n* = 5) and **g** for FGF7 (*n* = 6). **h** Recombinant CCL20, FGF7, EGF, but not IL15, significantly promoted the migration of parental HaCaT cells, as revealed by a wound healing assay. Bovine serum albumin (BSA) was used as a negative control. **i** Neutralization of CCL20 and FGF7 with specific antibodies abrogated the migration-promoting effect of BTG3-KO CM-Ad2. Migration of HaCaT cells was assayed as in **h**. IgG was used as a negative control antibody. **j, k** BTG3-KO CM-Ad2 promoted HaCaT spheroid formation in three-dimensional culture. HaCaT cells were seeded on Matrigel in the indicated CM-Ad2 **j** and the number of spheroids formed were counted and compared in **k**.

Mature adipocytes regulate tumor progression through adipokine secretion [31–33]. Given that BTG3 functions as a tumor suppressor [21, 34] and BTG3-KO CM and parental CM differed in their potential to promote 3T3-L1 adipogenic differentiation, we were intrigued by the possibility that the resulting adipocytes could be programmed differently for adipokine secretion. To explore this possibility, BTG3-KO and parental CM were collected in the early (Ad1) and late (Ad2) stages of adipocyte differentiation (Fig. 4a) and subjected to content analysis using cytokine antibody arrays. The levels of 16 cytokines and factors were found to be increased when comparing BTG3-KO CM-Ad2 to BTG3-KO CM-Ad1, some of which were preferentially elevated compared to their levels in parental CM (Fig. 4b). In particular, CCL20, IL15, FGF7, and EGF levels were significantly increased during BTG3-KO-mediated adipocyte differentiation (Fig. 4b). Importantly, these increases were abolished by the adipogenesis inhibitor genistein [35], suggesting they were produced by differentiating or differentiated adipocytes (Fig. 4c). In agreement with these in vitro observations, we also observed heightened CCL20 and FGF7 staining in the back skin tissue of *Btg3*^−/−^ mice compared to WT mice, indicating their physiological relevance (Fig. 4d–g).

In light of the differences in the contents of parental and BTG3-KO CM-Ads, we sought to determine whether differential behavioral impacts, for example, on cell migration, may be bestowed on keratinocytes via a feedback mechanism. To this end, we compared the migration of parental HaCaT cells in medium supplemented with either BSA (control) or recombinant human CCL20, IL15, FGF7, and EGF using a wound healing assay. The results indicated that, while IL15 had a less apparent effect, CCL20, FGF7, and EGF promoted HaCaT cell migration compared to the control treatment (Fig. 4h and Fig. S4a). In addition, we observed that, although neutralizing antibodies against CCL20 and FGF7 had no obvious impact on parental-CM-driven HaCaT cell migration, the antibodies significantly inhibited the migration-promoting effect of BTG3-KO CM (Fig. 4i and Fig. S4b). These results suggest that adipocytes differentiated in the milieu of BTG3-KO keratinocytes may affect keratinocyte migration through CCL20 and FGF7 secretion.

To determine if the two Ad2s affect HaCaT proliferation differently in three-dimensional organoid culture, we seeded HaCaT cells on Matrigel in either parental CM-Ad2 or BTG3-KO CM-Ad2. The results demonstrated that BTG3-KO CM-Ad2 promoted spheroid formation of the parental HaCaT cells (Fig. 4j and 4k). These data suggest that factors secreted by BTG3-KO CM-differentiated adipocytes could also promote HaCaT proliferation in an in vivo-mimicking condition.

To understand further the intrinsic difference between parental and BTG3-KO CM-differentiated adipocytes in their expression profiles, we prepared RNA from these differentiated adipocytes and performed RNA-seq analysis. Gene ontology (GO) analysis showed that the DEGs were most related to cellular function such as immune response, wound healing or cell migration, cell-substrate adhesion, angiogenesis, and interestingly, skin morphogenesis (Fig. S5a and Table S1). GSEA of DEGs revealed the enrichment of gene sets associated not only with adipogenesis [36] (Fig. S5b), but also with wound healing [37] (Fig. S5c) and TPA-induced mouse skin cancer [38] (Fig. S5d), thus further supporting our in vitro observation.

### BTG3 knockout in keratinocyte enhances cell migration and EMT

RNA-seq analysis comparing parental and BTG3-KO HaCaT cells revealed elevated expression in BTG3-KO cells of several signature genes, such as fibronectin and AKT3, which have been implicated in cancer cell migration and metastasis (Fig. S3a and Table S2). In addition, many genes associated with cell migration were also enriched by BTG3 knockout, as revealed by GSEA (Fig. S3b) [39] and GO analysis (Fig. S3c). To confirm this effect, EMT markers were examined in parental and BTG3-KO HaCaT cells using western blotting analysis. In line with the RNA-seq results, the levels of mesenchymal markers including Snail1, Slug, N-cadherin, vimentin, fibronectin, β-catenin, and ZO1 were increased in BTG3-KO cells. In contrast, the expression levels of E-cadherin, an epithelial marker, were downregulated (Fig. S3d). Consistent with the increase in the expression levels of EMT markers, BTG3 deletion in HaCaT cells promoted cell migration during wound healing (Fig. S3f and Fig. S6a) and in transwell migration assays (Fig. S3g), and this was not due to a difference in their proliferation (Fig. S3e). This effect on migration was mimicked by recombinant IL1α, IL10, or CCL4 (Fig. S3h and Fig. S6b), the same set of cytokines that stimulate adipogenesis (Fig. 2j), and was abolished by neutralizing antibodies targeting these factors (Fig. S3i and Fig. S6c). Moreover, we compared the autocrine effect with the paracrine from the reprogrammed adipocytes in promoting parental HaCaT migration. While Ad2 from parental HaCaT dampened the promoting effect of the respective CM, we did not observe such effect with BTG3 KO Ad2 on the respective CM (Fig. S7). In fact, both BTG3 KO CM and the respective Ad2 promoted HaCaT migration, although no further increase was seen when combined together (Fig. S7). Taken together, our results suggest that BTG3 loss affects keratinocyte migration not only through paracrine signaling of reprogrammed adipocytes but also through IL1α−, IL10−, and CCL4−mediated autocrine effects.

### BTG3 knockout promotes skin carcinogenesis

**Fig. 5:**
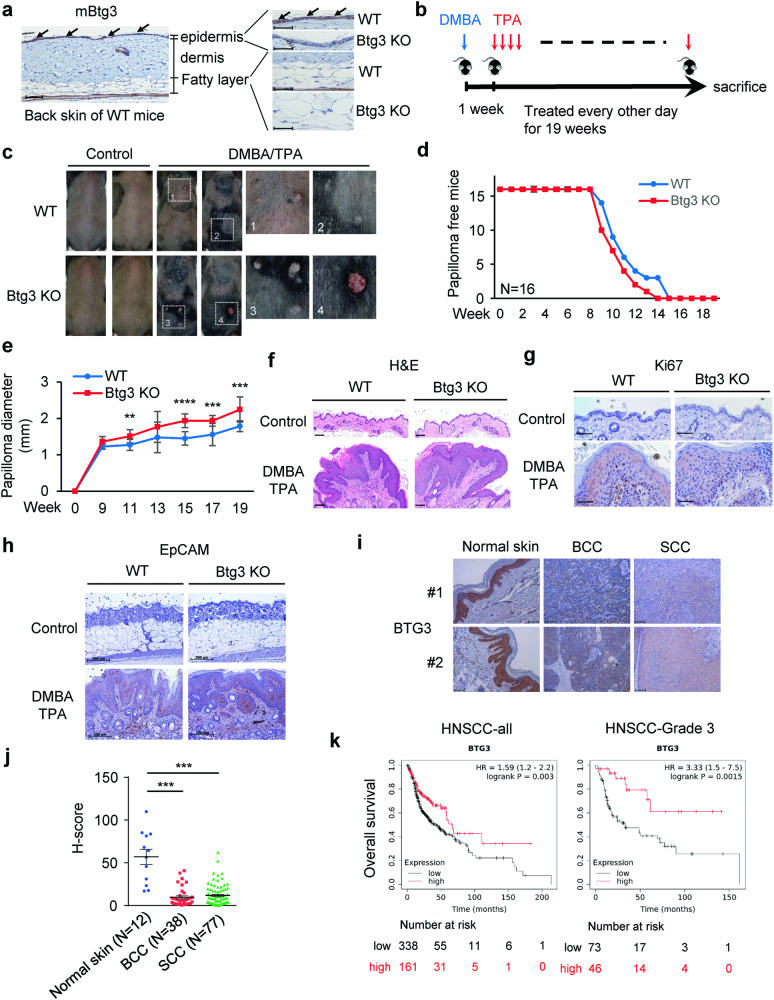
BTG3 ablation promotes skin cancer development. **a** Expression of Btg3 in the skin epidermis of WT but not *Btg3*^−/−^ mice, as revealed by IHC staining. **b** Workflow employed to generate the DMBA/TPA two-stage skin carcinogenesis model. **c** Skin papillomas at the treatment endpoint from control and DMBA/TPA-treated WT and *Btg3*^−/−^ mice. Acetone was used as a solvent control. Images 1 to 4 represent close-up views of areas 1 to 4, respectively, surrounding the papillomas. **d, e** Papillomas developed slightly faster and significantly larger in size in *Btg3*^−/−^ mice. Disease progression was monitored **d** and the average sizes of papillomas at the indicated time points were plotted and shown in **e**. Data were compiled from two independent experiments. **f** Hematoxylin and eosin staining of papillomas from the epidermis of control and DMBA/TPA-treated WT and *Btg3*^−/−^ mice. **g, h** IHC staining of the proliferation marker ki67 **g** and the basal cell carcinoma (BCC) marker EpCAM **h** in the epidermis of control and DMBA/TPA-treated WT and *Btg3*^−/−^ mice. **i, j** BTG3 is downregulated in human BCC and squamous cell carcinoma (SCC). IHC staining was performed on human skin cancer tissue microarrays with anti-BTG3 antibody **i**. Quantification of staining is shown in (j) as H scores. **k** Low BTG3 expression correlates with poorer patient survival in head and neck squamous cell carcinoma (HNSCC). Data were analyzed using Kaplan-Meier Plotter (http://kmplot.com/analysis/).

To understand the physiological relevance of our findings to skin-related pathology, we first determined the expression pattern of BTG3 in the skin. IHC staining of mouse skin showed that Btg3 was mainly expressed in the epidermis in WT mice, but not in *Btg3*^−/−^ mice that we have previously generated [22] (Fig. 5a). Similarly, skin cell type analysis using the Human Protein Atlas (https://www.proteinatlas.org) revealed that BTG3 was more abundantly expressed in basal keratinocytes than in many other cell types residing in the skin (Fig. S8).

BTG3 downregulation has been observed in many cancers, including renal, breast, and prostate cancer [18–20], but its role in non-melanoma skin cancer is unclear. To investigate whether BTG3 affects the development of skin cancer, a 7,12-dimethylbenz[a]anthracene (DMBA)/12-O-tetradecanoyl phorbol-13-acetate (TPA) two-stage skin carcinogenesis mouse model was employed [40] (Fig. 5b). The carcinogen DMBA was applied to the back skin of 8-week old WT and *Btg3*^−/−^ mice in the first week, and this was followed by 19 weeks of treatment with TPA only. We observed that papillomas developed and grew to a larger size on the skin of *Btg*3^−/−^ mice than on the skin of WT mice, suggesting that BTG3 loss may promote skin carcinogenesis (Fig. 5c–e). The difference in susceptibility became more pronounced in aged mice in which BTG3 KO mice had apparent thicker skin adipose tissue (Fig. S9a) and developed papillomas significantly earlier and larger in size than the WT (Fig. S9b and S9c). These papillomas were confirmed by hematoxylin and eosin, Ki67, and EpCAM staining (Fig. 5f–h, Fig. S9d and S9e) and did not show apparent pathological differences between WT and *Btg3*^−/−^ mice.

The results from the *Btg3*^−/−^ mice raised another question regarding the contribution of *Btg3*^−/−^ adipocytes in carcinogenesis. To address this issue, we first knocked out Btg3 in 3T3-L1 by CRISPR/Cas9. Two of the KO lines B1 and L1, which have one *Btg3* allele targeted and have reduced Btg3 expression (Fig. S10a and S10b), showed decreased cell viability (Fig. S10c) and colony formation (Fig. S10d), but still could be promoted to differentiate into adipocytes by BTG3 KO HaCaT CM (Fig. S10e). Furthermore, Ad2 collected from the differentiation assay using Btg3 KO 3T3-L1 could support parental HaCaT proliferation (Fig. S10f) and colony formation (Fig. S10g) indistinguishable from Ad2 of parental 3T3-L1. We conclude that BTG3 expression in adipocyte has minimal influence on its functional interaction with keratinocyte and likely on the outcome of carcinogenesis.

### BTG3 is downregulated in human non-melanoma skin cancers

To seek the association between BTG3 and clinical non-melanoma skin cancers, IHC staining of BTG3 was performed using human skin cancer tissue arrays. The results showed that the expression levels of BTG3 were significantly reduced in BCC and SCC compared to normal skin tissue (Fig. 5i and 5j). To determine the relationship with patient survival, we investigated the highly metastatic squamous cell carcinoma of the head and neck (HNSCC), which often exhibits high levels of NF-κB activity [41]. An analysis of the available datasets using the Kaplan–Meier Plotter (http://kmplot.com/analysis/) revealed that low BTG3 expression levels were associated with poor patient survival, especially in the later stages of HNSCC (Fig. 5k). Interestingly, higher expression ratios of BTG3 to IL1A, IL10, or CCL4 all correlated with better patient survival (Fig. S11). Collectively, these data support the notion that BTG3 functions as a tumor suppressor in non-melanoma skin cancer and likely in HNSCC.

## Discussion

**Fig. 6:**
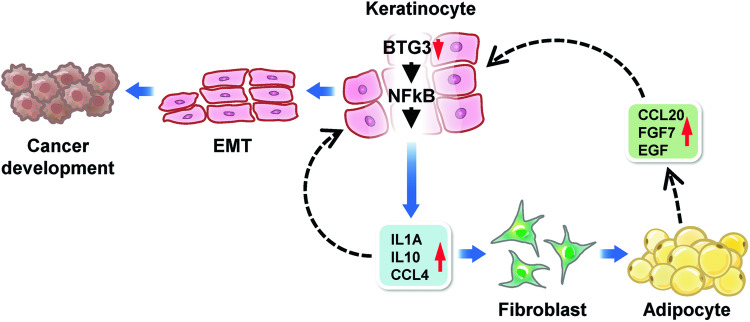
Model of BTG3 in the regulation of adipogenesis and the development of skin cancer. Our data are consistent with a role of BTG3 in safeguarding the functional interplay between keratinocytes and adipocytes. In the absence of BTG3, keratinocytes, by releasing IL1α, IL10 and CCL4, promote their own mesenchymal transition by an autocrine mechanism and adipocyte differentiation through paracrine. The latter, in turn, fuels further keratinocyte proliferation and migration by releasing EGF, CCL20, and FGF7, thus forming a feedforward loop to promote skin oncogenesis.

Here, we dissected the role of BTG3 in the functional interplay between keratinocytes and adipocytes, with a possible connection to neoplastic transition in skin cancer. Our data support a model in which BTG3-KO in keratinocytes promotes the NFκB-dependent release of IL1α, IL10, and CCL4. These cytokines/chemokines, on one hand, act in an autocrine fashion to enhance keratinocyte migration and possibly mesenchymal transition, and on the other hand, act in *trans* to promote adipocyte differentiation, during which secreted factors, such as CCL20, FGF7, and EGF, further augment the mesenchymal behavior of the keratinocytes (Fig. 6). Our findings highlight the importance of BTG3 in controlling functional interactions between keratinocytes and adipocytes in the skin. Skewing homeostasis, for example, by the deletion of BTG3, may lead to EMT and provide a window of opportunity for neoplastic transformation. Thus, these results provide a molecular link between programmed adipogenesis and cancer and showcase the role of BTG3 as a tumor suppressor by controlling the surrounding adipocytes.

Previous studies have shown that the hair follicle (HF) growth cycle is closely synchronized with oscillations in the thickness of the hypodermal adipose layer. When HFs grow deep into the adipose layer during the growth phase of the cycle, the thickness of the adipose layer increases significantly. There is evidence that the inhibition of Wnt/β-catenin-signaling-mediated Bmp2, Bmp6, and Igf2 secretion in epidermal keratinocytes reduces the adipose tissue in adult and embryonic skin [24]. However, it is unclear whether other signaling pathways are involved. Cancer cells release exosomes containing IL6 and miRNAs, which modify cancer-associated adipocytes [42, 43]. In turn, these cancer-associated adipocytes (CAA) undergo de-differentiation and lipolysis and further fuel tumor progression [44]. Although most studies have focused on the signaling events leading to adipocyte remodeling, the mechanism by which cancer cells gain the ability to reprogram adipocytes has been scarcely dissected. Unlike the interaction between existing cancer cells and CAA, here we present a different view on the early phase of neoplastic transition caused by loss of a tumor suppressor, and a unique regulatory loop between keratinocyte and adipocytes driving neoplastic progression in the skin.

Mechanistically, we showed that NF-κB may be the nexus of the regulatory activities of BTG3 in keratinocytes. We demonstrated that BTG3 reduced NF-κB activation by impacting p65 nuclear localization (Fig. 3d and 3e) and by obstructing its interaction with p300 (Fig. 3h). Similarly, we have previously shown that, by blocking the interaction between HIF1α and p300, BTG3 diminishes HIF1α acetylation and suppresses HIF1-mediated angiogenesis [22]. Taken together, these results suggest that BTG3 functions as an acetylation modulator that is involved in p300-mediated cellular regulation. In addition to acetylation, we have also reported that BTG3 promotes the CRL4^cdt2^-mediated K63-linked ubiquitination of CHK1 to safeguard genome stability [45]. The idea that BTG3 forms distinct complexes in cells that function in different post-translational regulatory mechanisms is certainly enticing, and warrants further investigation.

The functional interplay between BTG3 and NF-κB, as unraveled in this study, is interesting as the activity of NF-κB is very much associated with skin carcinogenesis, particularly in the DMBA/TPA model. Increased nuclear localization of p50 and p65 NF-κB has been observed in the skin tissue of mice treated with DMBA/TPA [46]. In addition, the TNF-α/NF-κB/Snail signaling pathway has long been known to contribute to cancer development and metastasis through a tumor-nurturing pro-inflammatory microenvironment [47]. p65-dependent NF-κB activation in epidermal keratinocytes is required for efficient DMBA/TPA-induced carcinogenesis [48]. In humans, highly metastatic HNSCC often exhibits a high level of NF-κB activity; and invasion by these cancer cells can be reduced by treatment with an NF-κB inhibitor [41]. Analogous with NF-κB inhibition, our results revealed that BTG3 may suppress the activity p65 by preventing its nuclear localization and interaction with the coactivator p300, thus preventing skin carcinogenesis. However, whether this relationship exists in other tissues or cancer types remains unclear. In fibroblasts, HIF-1α appears to assume the role of NF-κB and is directly regulated by BTG3. It is not unexpected that other regulatory targets may exist, and the mechanism of regulation in carcinogenesis may be tissue-specific.

Our study also illustrated an interesting regulatory feed-forward loop in which the same set of cytokines/chemokines that enhance keratinocyte migration in an autocrine fashion also remodel adipocytes to release adipokines that accentuate keratinocyte migration in a paracrine manner (Fig. 6). We showed that the elevated secretion of IL1α, IL10, and CCL4 by BTG3-KO keratinocytes, in addition to driving self-EMT (Fig. S3), promoted adipocyte differentiation (Fig. 2j). It is not unexpected that these cytokines/chemokines could promote keratinocyte migration, as previous studies have fully demonstrated their capability to do so in a variety of tissues and cancer types [49–55]. However, the roles of IL1, IL10, and CCL4 in adipocyte differentiation are less clear. A literature review revealed inconsistent conclusions. Although there have been reports suggesting a correlation between their increased levels and adipocyte differentiation [56–58], it is unclear whether these factors are directly involved in this process. Using recombinant factors and neutralizing antibodies, we provided direct evidence that, when used in combination, IL1α, IL10, and CCL4 significantly enhanced the differentiation of preadipocyte 3T3-L1 cells (Fig. 2j), suggesting that these factors may act as major proadipogenic factors in BTG3-KO CM. However, as each individual factor alone appeared to have a much smaller effect on differentiation (data not shown), it is likely that crosstalk among several signaling pathways may be required [59, 60].

Although we have focused on the consequence of BTG3 loss in skin carcinogenesis, the implications of our findings on cutaneous wound healing cannot be ignored. Our results suggest that dermal adipocytes may affect keratinocyte proliferation and migration or even the re-epithelialization of cutaneous wounds through secreted paracrine factors. Here, we showed that increased secretion of FGF7/keratinocyte growth factor, CCL20, and EGF by BTG3-KO-CM-stimulated adipocytes promoted keratinocyte migration, and the addition of neutralizing antibodies had the opposite effect (Fig. 4h and 4i). Although FGF7 and EGF are known to have such effects on keratinocytes [61, 62], these effects have not been reported for CCL20, despite that it has been shown previously to stimulate T-lymphocyte migration [63]. Indeed, in *Btg3*^−/−^ mice, we observed increased subcutaneous adipose tissue (Fig. 1a) and expanded basal layers in the epidermis (data not shown), suggesting that keratinocyte–adipocyte interactions may also exist in the skin. CCR6, the receptor for CCL20, is expressed in keratinocytes [64, 65], and the activity of NF-κB has been shown to mediate its downstream effects [66, 67]. In a BTG3-KO setting, CCL20 could drive a feedforward regulatory loop between keratinocytes and adjacent adipocytes, promoting abnormal keratinocyte proliferation and migration. However, in a WT background, CCL20 may promote wound healing without the risk of neoplastic transition. Whether and how this concept can be employed in a clinical setting warrant further investigation.

## Materials and methods

### Cell culture

Parental and BTG3-KO HaCaT cells (N7/B6/A3) were maintained in Dulbecco’s modified Eagle’s medium (DMEM; Gibco) supplemented with 10% fetal bovine serum (FBS), 100 U/mL penicillin, 100 μg/mL streptomycin, and 2 mM L-glutamine (all from Gibco). 3T3-L1 cells were maintained in DMEM supplemented with 10% calf serum (Gibco) and antibiotics. HEK293T cells were cultured in DMEM supplemented with 10% FBS and antibiotics.

### Generation of BTG3-KO HaCaT and 3T3-L1 cells

HaCaT and 3T3-L1 cells were transfected using Lipofectamine 2000 (Thermo Fisher Scientific, Waltham, MA, USA) with a plasmid (pZG12C03; ZGene Biotech Inc., Taiwan) expressing Cas9, GFP, and an sgRNA targeting human *BTG3* sequences (5´-TTGTGAAAAGAAGACAACGG-3´ [reverse strand]). GFP-positive cells were sorted by flow cytometry and further selected with 0.2 μg/mL puromycin to obtain stable clones. BTG3-KO clones were confirmed by immunoblotting (Fig. 1d and S10b), RT-qPCR (Fig. S10a) and genome sequencing (Fig. S1).

### 3T3-L1 adipocyte differentiation assay

CM was harvested from parental and BTG3-KO HaCaT cells cultured in complete medium for 2 d. 3T3-L1 cells were seeded in 12-well plates and allowed to reach full confluence after 2 d. The medium was then replaced with HaCaT CM plus 1 μM dexamethasone (D4902; Sigma) and 0.5 mM 3-isobutyl-1-methylxanthine (IBMX, I5879; Sigma) and incubated for 2 d to induce adipocyte differentiation. DMEM containing 10% FBS, 1 μg/mL insulin, 1 μM dexamethasone, and 0.5 mM IBMX was used as the positive control, and DMEM with 10% calf serum was used as the negative control. After induction, the cells were incubated with CM (or DMEM) containing 1 μg/mL insulin for 4 d and then with normal DMEM for another 2 d. Differentiated adipocytes were fixed in 4% paraformaldehyde after washing with PBS and stained with Oil Red O solution (O0625; Sigma) for 1 h. After washing with 60% isopropanol to remove the background, the Oil Red O stain was dissolved in 100% isopropanol and the absorbance was determined at 492 nm. Where indicated, genistein (G6649; Sigma Aldrich) was added in CM to final concentration of 100 μM.

### Immunofluorescence staining

For C/EBPα immunocytochemistry analysis, 3T3-L1 cells grown on coverslips were differentiated in CM from parental or BTG3 KO HaCaT cells. After adipogenesis, cells were fixed in 4% paraformaldehyde/PBS at room temperature for 20 min, permeabilized in 0.5% Triton X-100/PBS on ice for 10 min. and blocked in 5% BSA/PBS at room temperature for 60 min. Cells were stained with anti-C/EBPα antibody and then with FITC-conjugated secondary antibody (Jackson ImmunoResearch). The C/EBPα staining was imaged using Zeiss LSM700 stage confocal microscope and quantified using ZEN 3.5 software (Carl Zeiss, Oberkochen, Germany).

### Three-dimensional culture

Parental and BTG3 KO HaCaT cells were trypsinized and resuspended in DMEM complete medium (BD Biosciences) containing 2% Matrigel (BD Biosciences). Five thousand cells were seeded into each well of an 8-well chamber slide (Millipore) precoated with 100 μl of Matrigel. After one day, the culture medium was changed to Ad2 from HaCaT CM-induced adipogenesis and refreshed every 3 days until the day of assay. For fluorescence staining of the 3-D culture, the spheroids were fixed in chamber slides with 4% paraformaldehyde for 30 min at room temperature. After permeabilization in 0.5% Triton X-100/PBS on ice for 10 min and staining with DAPI, the images were captured using a fluorescence microscope system (Zeiss Axiovert 200 M).

### Co-culture

Ten thousand 3T3-L1 cells were plated on the lower chamber of a 48-well dish in DMEM and 5000 parental and BTG3 knockout HaCaT cells were seeded in the hanging inserts with pore size of 0.4 μm (PTHT24H48; Millipore) at the same time. After 2 days of incubation, 1 μM dexamethasone (D4902; Sigma) and 0.5 mM 3-isobutyl-1-methylxanthine (IBMX) (I5879; Sigma) were added and regular differentiation protocol was followed. Adipogenesis was determined by Oil Red O staining.

### Plasmids

The pGL4.32 [luc2P/NF-κB-RE/Hygro] vector (Promega) that contains five copies of an NF-κB response element was used for the luciferase reporter assay. The cDNA encoding human p65 was amplified from HEK293T cells by PCR and cloned in between the *Bam*HI and *Xho*I sites of the pXJN-HA vector^34^. Constructs expressing BTG3 have been previously described [45].

### RNA interference

All siRNA was synthesized by Sigma-Aldrich and transfected using Oligofectamine (Invitrogen) for 2 d before analysis. Sequences targeted by BTG3 and RELA siRNA were 5´-TTGAGAGGTTTGCTGAGAA-3´ and 5´-CCTTTCTCATCCCATCTTT-3´, respectively.

### Cell lysis and immunoblotting

Cell lysates were prepared in TEGN buffer (10 mM Tris [pH 7.5], 1 mM EDTA, 420 mM NaCl, 10% glycerol, and 0.5% Nonidet P-40) containing 10 mM NaF, 10 mM glycerophosphate, 1 mM sodium orthovanadate, 1 mM dithiothreitol, and a protease inhibitor cocktail (Roche). For immunoblotting analysis, the proteins were separated by sodium dodecyl sulfate polyacrylamide gel electrophoresis (SDS-PAGE), transferred to a nitrocellulose membrane, and detected using specific antibodies. The signals were detected using chemiluminescent reagents (NEL105001EA; Perkin Elmer) and captured using GeneGnome (Syngene).

### Coimmunoprecipitation

For the coimmunoprecipitation of proteins overexpressed in HEK293T cells, cell lysates prepared in TEGN buffer were diluted with TEG buffer (10 mM Tris, pH 7.5, 1 mM EDTA, and 20% glycerol) and incubated with antibodies and protein A resins (Thermo Fisher Scientific). After rocking for 2 h at 4°C, the beads were washed three times in the same buffer, boiled in protein sample buffer, and analyzed by SDS-PAGE.

### Antibodies and recombinant proteins

All antibodies used are listed in Table S3. The rabbit anti-BTG3 antibody has been described previously [34].

The following recombinant proteins were used: IL1α (200-LA-002), IL10 (217-IL-005), CCL4 (271-BME-010), CCL20 (360-MP-025), FGF7 (251-KG-010), and IL15 (247-ILB-005) from R&D Systems, and EGF (E4269) from Sigma-Aldrich.

### Cytokine antibody array analysis

CM was prepared as indicated and stored at −80 °C before use. The analysis was conducted using a human cytokine antibody array (RayBio® C-Series Human Cytokine Antibody Array C1000; RayBiotech) following the manufacturer’s protocol. The intensity of the signals was quantified using MetaMorph software.

### DMBA/TPA two-stage skin carcinogenesis model

The protocol described by Abel et al. [40] was followed, with slight modifications, to construct the skin carcinogenesis model. Ten 8-week old WT and *Btg3*^−^^/^^−^ mice [22] were shaved on the back 2 d before treatment with the initiator DMBA (D3254; Sigma) at 25 nmol per mouse in 200 μl of acetone. One week later, the back skin of each mouse was treated three times weekly with the tumor promoter TPA (P8139; Sigma) at 6.5 nmol per mouse in 200 μL of acetone for 19 weeks. Acetone-only treatment was used as a control. The incidence and size of papillomas were recorded weekly. The animal studies were conducted following the guidelines and was approved by the Institutional Animal Care and Utilization Committee of Academia Sinica (AS IACUC, Protocol ID 11-12-271).

### Transwell migration assay

Cell migration was assayed in a 24-well Boyden chamber (Costar). Equal numbers of parental and BTG3-KO HaCaT cells were plated in serum-free medium in the upper compartment of a Boyden chamber, with the lower compartment containing 700 μL of complete medium. After 24 h of incubation, cells in the upper chamber were fixed with 100% methanol and stained with crystal violet. The cells on the lower surface of each insert in five random objective fields were counted using a light microscope.

### Immunohistochemistry

Human skin cancer tissue microarrays (T211a, SK484, and SK802b; US Biomax Inc.) were treated with 3% H_2_O_2_ after antigen retrieval. The arrays were then stained with an anti-BTG3 antibody (1:100) at 4 °C overnight, followed by a horseradish-peroxidase-conjugated polymer anti-rabbit antibody (K4003; Dako) for 1 h at room temperature. After development with 3,3’-diaminobenzidine substrate (K3467; Dako) and counterstaining with hematoxylin, images were captured using a Zeiss Imager A1 microscope (Zeiss).

Anti-ki67, −EpCAM, −CCL20, −FGF7, −IL1α, −IL10, and −CCL4 antibodies were used for IHC staining of mouse skin. All slides were scanned using a Slide Scanner Pannoramic 250 FLASH II instrument (3DHISTECH Ltd.) and analyzed using Pannoramic Viewer software. NuclearQuant was used to visualize the positively stained cells and determine their staining intensities.

### RT-qPCR

Total RNA was extracted from parental, BTG3-KO, and transfected HaCaT cells using TRIzol (15596018; Thermo Fisher Scientific) and reverse transcribed into cDNA using an oligo-dT primer (18418012; Thermo Fisher Scientific) and SuperScript IV reverse transcriptase (18090010; Thermo Fisher Scientific). The primers used for qPCR are listed in Table S4.

qPCR reactions were conducted in a 30 μL reaction mix in a 96-well plate using Power SYBR™ Green PCR Master Mix (REF4367659; Applied Biosystems). Reactions were carried out using an ABI 7500 system (Applied Biosystems), and relative expression values were calculated using the ABI 7500 software. The housekeeping gene, β-actin, was used as an internal control. The expression level of each gene was defined based on the threshold cycle (Ct), and the relative expression levels were calculated using the 2^−^^ΔΔCt^ method.

### Enzyme-linked immunosorbent assays

For enzyme-linked immunosorbent assays (ELISAs), CM were collected as described for the adipogenesis assay, and the concentrations of CCL20, FGF7, and gp130 were measured according to the procedure recommended by the kits’ manufacturer (EHCCL20, EHFGF7, and EHIL6ST; Thermo Fisher Scientific).

### Statistical analysis

All values, expressed as mean ± standard deviation, were obtained from at least three independent experiments, unless indicated otherwise. Significance was determined using the Student’s *t*-test or the methods specified in the figure legends. Differences were considered statistically significant at *P* < 0.05.

### Supplementary information

Table S1

Table S2

Supplemental information

Original data files

## Data Availability

Materials used in this study are available upon request. DEGs from RNA-seq have been compiled and are exhibited as Table S1 and Table S2.
