# Supplementary material for: A keratinocyte-adipocyte signaling loop is reprogrammed by loss of BTG3 to augment skin carcinogenesis
Source: Cell Death Differ. 2024 May 7;31(8):970–82. doi: 10.1038/s41418-024-01304-7 (PMC11303697; doi:10.1038/s41418-024-01304-7)
Supplement: Supplementary file 1 — Table S1 [file 41418_2024_1304_MOESM1_ESM.pdf]

Table S1. DEGs in parental and BTG3 KO CM differentiated adipocytes ($p<0.05$)

ensembl_gene_id	Gene name	symbol	log2 (CM-BTG3 KO/Parental)	pvalue
ENSMUSG00000106106			1.139977731	0
ENSMUSG00000035202	leucyl-tRNA synthetase, mitochondrial	Lars2	0.93385375	2.47E-39
ENSMUSG00000076258	predicted gene, 23935	Gm23935	0.911995827	4.401E-32
ENSMUSG00000092341	metastasis associated lung adenocarcinoma transcript 1	Malat1	0.433339122	3.012E-28
ENSMUSG00000029661	collagen, type I, alpha 2	Col1a2	-0.128781425	1.448E-07
ENSMUSG00000001506	collagen, type I, alpha 1	Col1a1	-0.184022239	2.074E-07
ENSMUSG00000070327	ring finger protein 213	Rnf213	-0.211018521	3.784E-07
ENSMUSG00000026043	collagen, type III, alpha 1	Col3a1	-0.14057018	7.758E-07
ENSMUSG00000021831	ERO1-like (S. cerevisiae)	Ero1l	0.202320836	0.0001027
ENSMUSG00000074896	interferon-induced protein with tetratricopeptide repeats 3	Ifit3	-0.404073395	0.0001888
ENSMUSG00000029838	pleiotrophin	Ptn	-0.159974164	0.0004389
ENSMUSG00000026473	glutamate-ammonia ligase (glutamine synthetase)	Glul	0.453600015	0.0015833
ENSMUSG00000000386	MX dynamin-like GTPase 1	Mx1	-0.576570258	0.0025451
ENSMUSG00000056025	chloride channel accessory 3A1	Clca3a1	0.205316101	0.0025886
ENSMUSG00000040856	delta like non-canonical Notch ligand 1	Dlk1	-0.128983768	0.0031613
ENSMUSG00000034459	interferon-induced protein with tetratricopeptide repeats 1	Ifit1	-0.327852091	0.0031973
ENSMUSG00000064351	mitochondrially encoded cytochrome c oxidase I	mt-Co1	-0.082072793	0.0045292
ENSMUSG00000029580	actin, beta	Actb	-0.136487431	0.004765
ENSMUSG00000040152	thrombospondin 1	Thbs1	-0.196049823	0.0076545
ENSMUSG00000037411	serine (or cysteine) peptidase inhibitor, clade E, member 1	Serpine1	0.321345526	0.009358
ENSMUSG00000038393	thioredoxin interacting protein	Txnip	0.20806356	0.0116498
ENSMUSG00000002020	latent transforming growth factor beta binding protein 2	Ltbp2	-0.238468758	0.0122442
ENSMUSG00000069793	schlafen 9	Slfn9	-0.40926464	0.0143716
ENSMUSG00000062488	interferon-induced protein with tetratricopeptide repeats 3B	Ifit3b	-0.388433227	0.0210124
ENSMUSG00000033880	lectin, galactoside-binding, soluble, 3 binding protein	Lgals3bp	-0.17125451	0.0228143
ENSMUSG00000069833	AHNAK nucleoprotein (desmoyokin)	Ahnak	0.067731315	0.025045
ENSMUSG00000031328	filamin, alpha	Flna	-0.105214433	0.0269515
ENSMUSG00000064370	mitochondrially encoded cytochrome b	mt-Cytb	-0.106246941	0.0287597
ENSMUSG00000003617	ceruloplasmin	Cp	0.175450976	0.0379377
ENSMUSG00000086583	predicted pseudogene 15500	Gm15500	-0.708266992	0.0381288
ENSMUSG00000027204	fibrillin 1	Fbn1	-0.217511082	0.0410967
ENSMUSG00000022587	lymphocyte antigen 6 complex, locus E	Ly6e	-0.300067829	0.0431044
ENSMUSG00000069874	immunity-related GTPase family M member 2	Irgm2	-0.309279809	0.0442381

ENSMUSG00000035692	ISG15 ubiquitin-like modifier	Isg15	-0.354040855	0.0487342
