# Supplementary material for: A keratinocyte-adipocyte signaling loop is reprogrammed by loss of BTG3 to augment skin carcinogenesis
Source: Cell Death Differ. 2024 May 7;31(8):970–82. doi: 10.1038/s41418-024-01304-7 (PMC11303697; doi:10.1038/s41418-024-01304-7)
Supplement: Supplementary file 2 — Table S2 [file 41418_2024_1304_MOESM2_ESM.pdf]

Table S2. DEGs in parental and BTG3 KO HaCaT (Fold change 2, p<0.005)

ensembl_gene_id	symbol	description	log2 (BTG3 KO/Parental)	pvalue
ENSG00000115414	FN1	fibronectin 1	1.444107789	3.58E-106
ENSG00000157657	ZNF618	zinc finger protein 618	3.712301347	1.693E-20
ENSG00000117020	AKT3	AKT serine/threonine kinase 3	5.48214195	2.988E-19
ENSG00000150687	PRSS23	protease, serine 23	1.390336087	7.872E-19
ENSG00000224078	SNHG14	small nucleolar RNA host gene 14	2.859644543	6.461E-16
ENSG00000101210	EEF1A2	eukaryotic translation elongation factor 1 alpha 2	2.102314132	2.235E-15
ENSG00000169047	IRS1	insulin receptor substrate 1	2.014327831	1.821E-11
ENSG00000113657	DPYSL3	dihydropyrimidinase like 3	1.114563807	2.707E-11
ENSG00000179820	MYADM	myeloid-associated differentiation marker	1.696554673	1.873E-10
ENSG00000176788	BASP1	brain abundant membrane attached signal protein 1	1.332457453	8.481E-10
ENSG00000177707	PVRL3		4.261973198	9.987E-09
ENSG00000143702	CEP170	centrosomal protein 170	1.148711939	2.453E-08
ENSG00000122786	CALD1	caldesmon 1	1.514818412	4.126E-08
ENSG00000000971	CFH	complement factor H	2.153416885	5.023E-08
ENSG00000163513	TGFBR2	transforming growth factor beta receptor 2	1.051864866	7.293E-08
ENSG00000132970	WASF3	WAS protein family member 3	3.586376292	5.474E-07
ENSG00000089250	NOS1	nitric oxide synthase 1	2.342159044	6.674E-07
ENSG00000152495	CAMK4	calcium/calmodulin-dependent protein kinase IV	3.726863907	6.862E-07
ENSG00000185070	FLRT2	fibronectin leucine rich transmembrane protein 2	3.76578203	8.662E-07
ENSG00000148677	ANKRD1	ankyrin repeat domain 1	2.069433215	1.424E-06
ENSG00000154678	PDE1C	phosphodiesterase 1C	3.219613243	3.088E-06
ENSG00000196159	FAT4	FAT atypical cadherin 4	3.394114961	4.541E-06
ENSG00000173517	PEAK1	pseudopodium enriched atypical kinase 1	2.20634316	5.141E-06
ENSG00000144354	CDCA7	cell division cycle associated 7	1.159713865	8.72E-06
ENSG00000151632	AKR1C2	aldo-keto reductase family 1, member C2	1.144523808	9.1E-06
ENSG00000198846	TOX	thymocyte selection associated high mobility group box	3.958051958	1.11E-05
ENSG00000233251	AC007743		2.142381905	1.276E-05
ENSG00000164574	GALNT10	polypeptide N-acetylgalactosaminyltransferase 10	1.590100618	1.829E-05
ENSG00000115355	CCDC88A	coiled-coil domain containing 88A	1.471477505	1.995E-05
ENSG00000169851	PCDH7	protocadherin 7	1.168952952	2.032E-05
ENSG00000197457	STMN3	stathmin 3	1.343121059	4.415E-05
ENSG00000106070	GRB10	growth factor receptor bound protein 10	2.068732048	5.339E-05
ENSG00000048342	CC2D2A	coiled-coil and C2 domain containing 2A	2.675576464	5.438E-05

ENSG00000181104	F2R	coagulation factor II thrombin receptor	1.587765235	6.915E-05
ENSG00000113758	DBN1	drebrin 1	2.881576288	7.099E-05
ENSG00000135919	SERPINE2	serpin family E member 2	1.040213364	7.336E-05
ENSG00000130707	ASS1	argininosuccinate synthase 1	-1.548243268	1.355E-40
ENSG00000196730	DAPK1	death associated protein kinase 1	-2.942432923	1.059E-29
ENSG00000183742	MACC1	metastasis associated in colon cancer 1	-1.198140109	5.834E-23
ENSG00000108691	CCL2	C-C motif chemokine ligand 2	-1.547419083	1.897E-19
ENSG00000189334	S100A14	S100 calcium binding protein A14	-1.204977352	1.269E-17
ENSG00000163283	ALPP	alkaline phosphatase, placental	-1.323059147	1.432E-11
ENSG00000197632	SERPINB2	serpin family B member 2	-1.063509691	5.338E-11
ENSG00000117472	TSPAN1	tetraspanin 1	-1.774291254	1.288E-08
ENSG00000197641	SERPINB13	serpin family B member 13	-1.237547499	1.899E-08
ENSG00000126016	AMOT	angiomotin	-1.507191431	3.638E-08
ENSG00000102081	FMR1	fragile X mental retardation 1	-1.036337893	4.652E-08
ENSG00000166396	SERPINB7	serpin family B member 7	-1.041557322	1.114E-07
ENSG00000143126	CELSR2	cadherin EGF LAG seven-pass G-type receptor 2	-1.343118124	1.389E-07
ENSG00000277586	NEFL	neurofilament, light polypeptide	-1.037648667	2.7E-07
ENSG00000148344	PTGES	prostaglandin E synthase	-1.043319365	5.183E-07
ENSG00000167754	KLK5	kallikrein related peptidase 5	-1.325629125	1.069E-06
ENSG00000142798	HSPG2	heparan sulfate proteoglycan 2	-1.534957292	1.092E-06
ENSG00000140284	SLC27A2	solute carrier family 27 member 2	-1.171878654	6.193E-06
ENSG00000171208	NETO2	neuropilin and tolloid like 2	-1.653056412	6.558E-06
ENSG00000161921	CXCL16	C-X-C motif chemokine ligand 16	-1.037854604	1.104E-05
ENSG00000136689	IL1RN	interleukin 1 receptor antagonist	-1.639036528	1.594E-05
ENSG00000169174	PCSK9	proprotein convertase subtilisin/kexin type 9	-1.432198119	2.202E-05
ENSG00000268621	AC006262		-1.325558874	2.519E-05
ENSG00000128833	MYO5C	myosin VC	-1.595274645	5.693E-05
ENSG00000105357	MYH14	myosin, heavy chain 14, non-muscle	-1.073857826	6.289E-05
ENSG00000130066	SAT1	spermidine/spermine N1-acetyltransferase 1	-1.248546107	6.426E-05
ENSG00000149328	GLB1L2	galactosidase beta 1 like 2	-1.16510534	6.394E-05
