# Supplementary material for: A keratinocyte-adipocyte signaling loop is reprogrammed by loss of BTG3 to augment skin carcinogenesis
Source: Cell Death Differ. 2024 May 7;31(8):970–82. doi: 10.1038/s41418-024-01304-7 (PMC11303697; doi:10.1038/s41418-024-01304-7)
Supplement: Supplementary file 3 — Supplemental information [file 41418_2024_1304_MOESM3_ESM.pdf]

Supplemental Information

A keratinocyte-adipocyte signaling loop is reprogrammed by loss of BTG3 to augment skin carcinogenesis

Cheng et al.

Supplemental Table S3 and S4

Figures and Figure Legends

Figure S1 Related to Figure 1

Figure S2 Related to Figure 1

Figure S3

Figure S4 Related to Figure 4

Figure S5 Related to Figure 4

Figure S6 Related to Figure S3

Figure S7 Related to Figure S3 and Figure 4

Figure S8 Related to Figure 5

Figure S9 Related to Figure 5

Figure S10 Related to Figure 5

Figure S11 Related to Figure 5

Table S3. List of Antibodies

Antibodies	Source	Identifier	Application
Acetyl-NFkB p65 (Lys310)	Invitrogen	PA5-17264	IP
Actin	Sigma-Aldrich	A2066	WB
α-tubulin	Sigma-Aldrich	T6074	WB
β--catenin	BD Biosciences	610154	WB
CCL4	LSBio	LS-C89548-100	IHC
	R&D	MAB271-100	Neutralization
CCL20	Bioss Inc.	bs-1268R	IHC, Neutralization
C/EBPα	BioLegend	662102	ICC
E-cadherin	Cell Signaling	#3195	WB
EpCAM	Merck	SAB4200690	IHC
FGF7	Bioss Inc.	bs-0734R	IHC, Neutralization
Fibronectin	Sigma-Aldrich	F6140	WB
Flag	LTK BioLaboratories		WB
GST	Santa Cruz	sc-138	WB
HA	LTK BioLaboratories		IP
	LTK BioLaboratories		WB
IL10	Bioss Inc.	bs-0698R	IHC
	R&D	AF-217-NA	Neutralization
IL1α	GeneTex	GTX74157	IHC
	R&D	AF-200-NA	Neutralization
Ki67	Novus	NB110-89719	IHC
Lamin A/C	Santa Cruz	sc-7292	WB
myc	Santa Cruz	sc-40	WB
N-cadherin	Cell Signaling	#4061	WB
NFκB p65	Cell Signaling	#6956	WB
Slug	Cell Signaling	#9585	WB
Snail1	Cell Signaling	#3879	WB
Vimentin	Cell Signaling	#3932	WB
ZO1	Cell Signaling	#5406	WB

Table S4. List of primers for RT-qPCR

Gene	Primer Pairs (5' to 3')
IL1A	ATCAGTACCTCACGGCTGC<br>GCATCTCCTTCAGCAGCAC
IL10	GGTTGCCAAGCCTTGTCTG<br>AATCGATGACAGCGCCGTA
CCL4	TGAAGCTCTGCGTGACTGT<br>AGGCTGCTGGTCTCATAGT
VEGFD	GCATCCCATCGGTCCACTA<br>TTGCAACAGCCACCACATC
XCL1	AAGAGGACCTGTGTGAGCC<br>CTGTCCATGCTCCTGACCA
CCL25	ACAGGAAGGTGTGTGGGAA<br>CTGCTGCTGATGGGATTGC
IL13	ACCACGGTCATTGCTCTCA<br>CATGCCAGCTGTCAGGTTG
IL5	GTGTATGCCATCCCCACAGA<br>TTCTTCAGTGCACAGTTGGT
CCL7	CTTCTGTGTCTGCTGCTCAC<br>ATTACAGCTTCCCGGGGAC
RELA	CCCACCATCAAGATCAATG<br>TTCTGGAAACTGTGGATGC
ACTIN	CCAGAGCAAGAGAGGCATCC<br>GTGGTGGTGAAGCTGTAGCC
mCebpa	CGGCGGTGACTTTGACTAC<br>GTGGCTGGTAGGGGAAGAG
mPparg	CAGAAGTGCCTTGCTGTGG<br>TCCTGTCAAGATCGCCCTC
mGapdh	CAACTCCCACTCTTCCACCT<br>CTTGCTCAGTGTCCTTGCTG

a

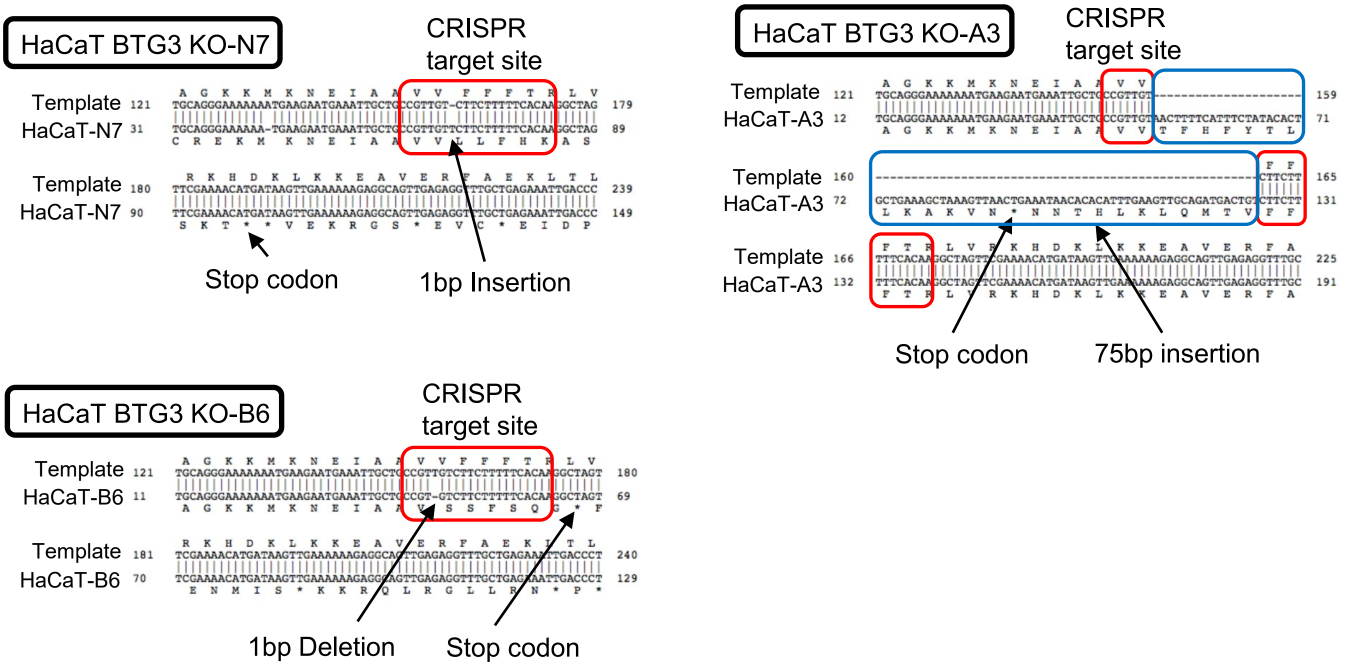

Figure S1. Confirmation of BTG3 gene ablation in the three knockout (KO) HaCaT clones by genome sequencing. The sequence and coding changes are shown for clones N7, A3, and B6, which generated premature translation termination.

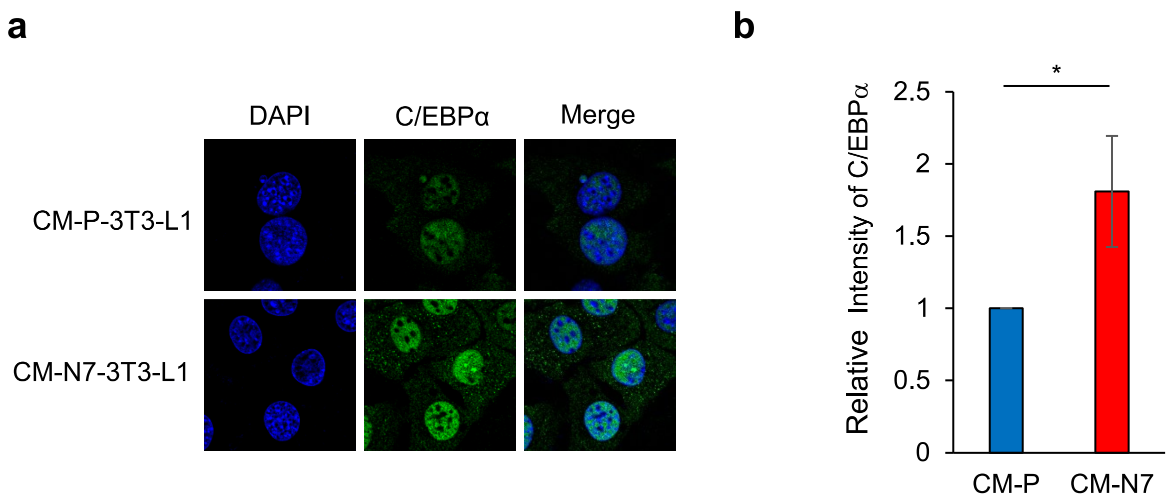

Figure S2. BTG3-KO keratinocytes promote the expression of C/EBPα adipogenic differentiation of 3T3-L1. Immunofluorescence staining was performed on differentiating 3T3-L1 2 days after the addition of indicated CM and insulin using anti-C/EBP antibody **(a).** The staining intensity was quantified and compared between parental and BTG3-KO CM **(b).**

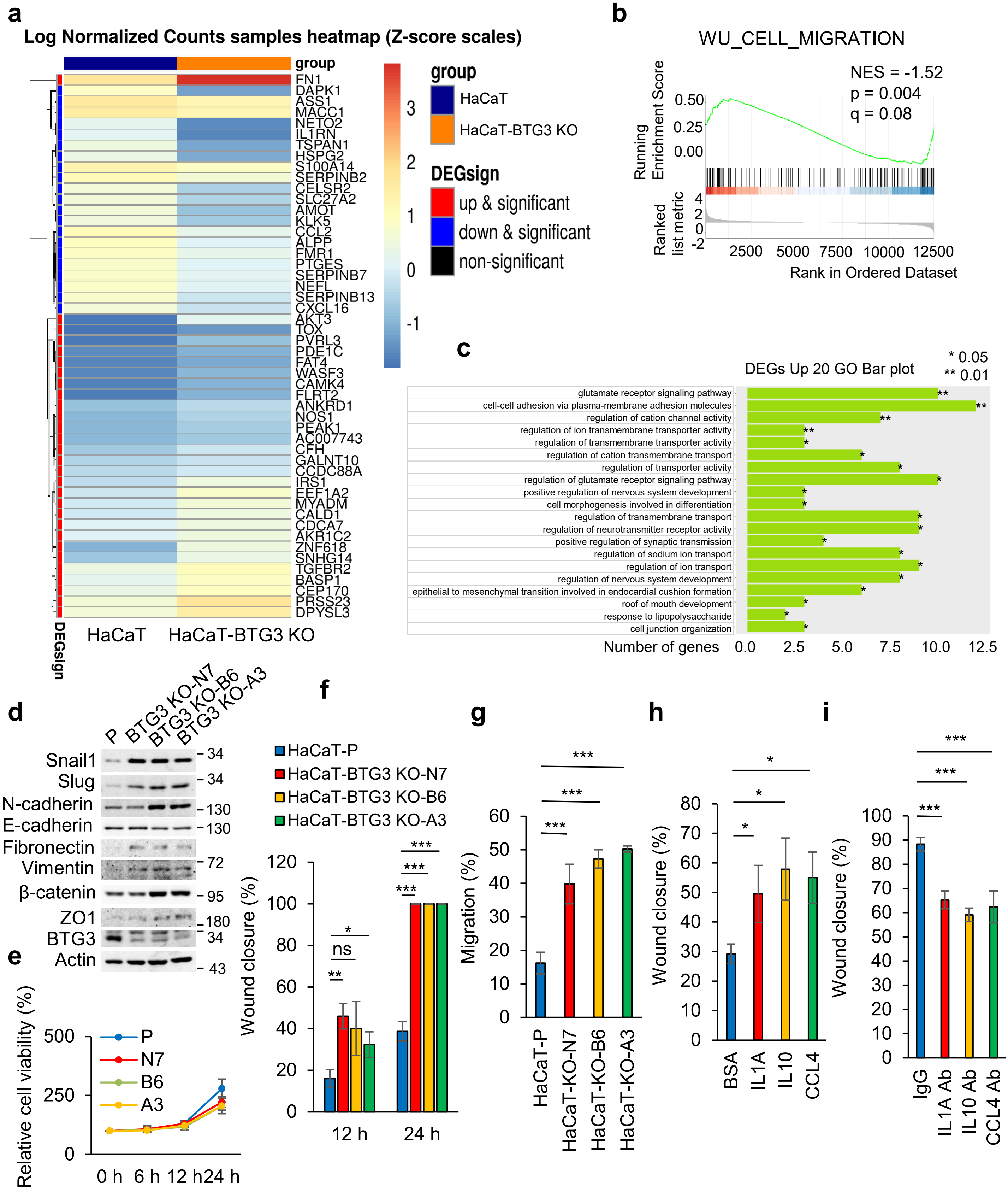
a
Log Normalized Counts samples heatmap (Z-score scales)
group
FN1
DAPK1
ASS1
MACC1
NETO2
IL1RN
TSPAN1
HSPG2
S100A14
SERPINB2
CELSR2
SLC27A2
AMOT
KLK5
CCL2
ALPP
FMR1
PTGES
SERPINB7
NEFL
SERPINB13
CXCL16
AKT3
TOX
PVRL3
PDE1C
FAT4
WASF3
CAMK4
FLRT2
ANKRD1
NOS1
PEAK1
AC007743
CFH
GALNT10
CCDC88A
IRS1
EEF1A2
MYADM
CALD1
CDCA7
AKR1C2
ZNF618
SNHG14
TGFBR2
BASP1
CEP170
PRSS23
DPYSL3
DEGsign
HaCaT
HaCaT-BTG3 KO
group
HaCaT
HaCaT-BTG3 KO
DEGsign
up & significant
down & significant
non-significant
b
WU_CELL_MIGRATION
NES = -1.52
p = 0.004
q = 0.08
Running Enrichment Score
Ranked list metric
Rank in Ordered Dataset
c
DEGs Up 20 GO Bar plot
* 0.05
** 0.01
glutamate receptor signaling pathway
cell-cell adhesion via plasma-membrane adhesion molecules
regulation of cation channel activity
regulation of ion transmembrane transporter activity
regulation of transmembrane transporter activity
regulation of cation transmembrane transport
regulation of transporter activity
regulation of glutamate receptor signaling pathway
positive regulation of nervous system development
cell morphogenesis involved in differentiation
regulation of transmembrane transport
regulation of neurotransmitter receptor activity
positive regulation of synaptic transmission
regulation of sodium ion transport
regulation of ion transport
regulation of nervous system development
epithelial to mesenchymal transition involved in endocardial cushion formation
roof of mouth development
response to lipopolysaccharide
cell junction organization
Number of genes
d
P
BTG3 KO-N7
BTG3 KO-B6
BTG3 KO-A3
Snail1
Slug
N-cadherin
E-cadherin
Fibronectin
Vimentin
β-catenin
ZO1
BTG3
Actin
e
Relative cell viability (%)
P
N7
B6
A3
0 h
6 h
12 h
24 h
f
HaCaT-P
HaCaT-BTG3 KO-N7
HaCaT-BTG3 KO-B6
HaCaT-BTG3 KO-A3
Wound closure (%)
ns
12 h
24 h
g
Migration (%)
HaCaT-P
HaCaT-KO-N7
HaCaT-KO-B6
HaCaT-KO-A3
h
Wound closure (%)
BSA
IL1A
IL10
CCL4
i
Wound closure (%)
IgG
IL1A Ab
IL10 Ab
CCL4 Ab

Figure S3. BTG3 KO in keratinocytes promotes migration and epithelial-mesenchymal transition (EMT) through an autocrine mechanism. **(a)** Heatmap of differentially expressed genes (DEGs) from RNA-seq analysis of parental and BTG3-KO HaCaT cells. **(b)** Gene set enrichment analysis (GSEA) showing that the up-regulated genes in BTG3-KO cells are enriched in processes related to cell migration. **(c)** Gene ontology (GO) analysis showing that the up-regulated genes in BTG3-KO cells are most associated with cell-cell adhesion and EMT. **(d)** Expression of several EMT markers was elevated in BTG3-KO HaCaT cells, as determined by western blot analysis. **(e)** Cell proliferation was comparable between parental HaCaT cells and the three BTG3 KO clones, N7, B6, and A3. **(f, g)** Cell migration was enhanced by the loss of BTG3. Wound healing (f) and transwell migration (g) were assessed with parental and the three BTG3-KO HaCaT cells. **(h)** Recombinant IL1 , IL10, and CCL4 promoted parental HaCaT cell migration, as analyzed by wound healing assays. **(i)** Neutralizing antibodies for IL1α, IL10, and CCL4 reduced the migration of BTG3-KO HaCaT cells, as revealed by wound healing assays.

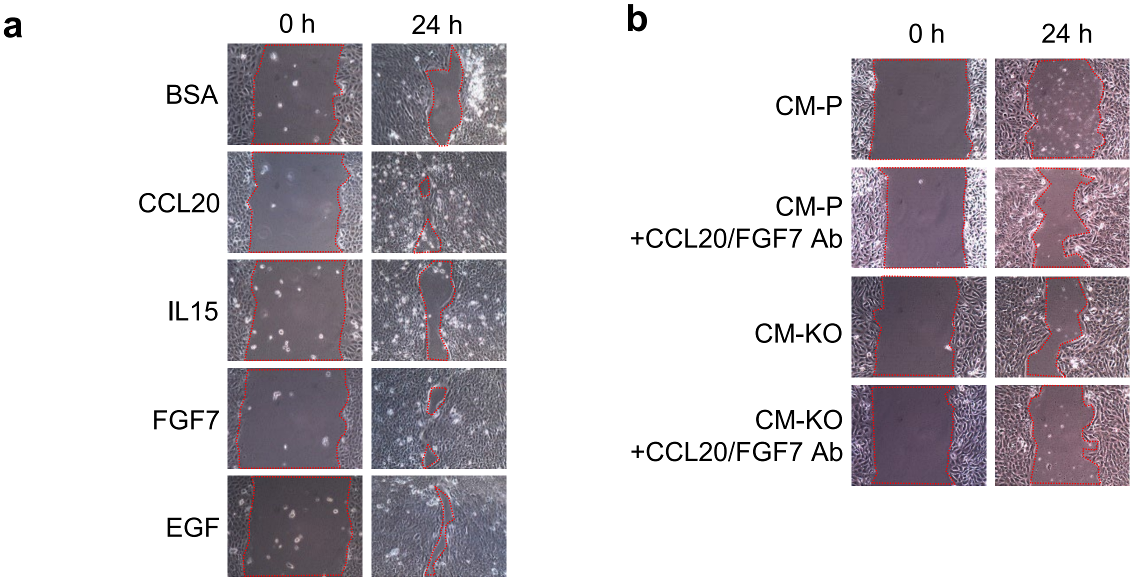

Figure S4. CCL20 and FGF7 in the BTG3-KO CM-Ad2 promote HaCaT migration. **(a)** Recombinant CCL20, FGF7, EGF, but not IL15 promoted the migration of parental HaCaT cells in wound healing. Images correspond to the results shown in Fig. 4h. **(b)** Neutralization of CCL20 and FGF7 with specific antibodies abrogated the migration-promoting effect of BTG3-KO CM-Ad2. Images are in support of the results shown in Fig.4i.

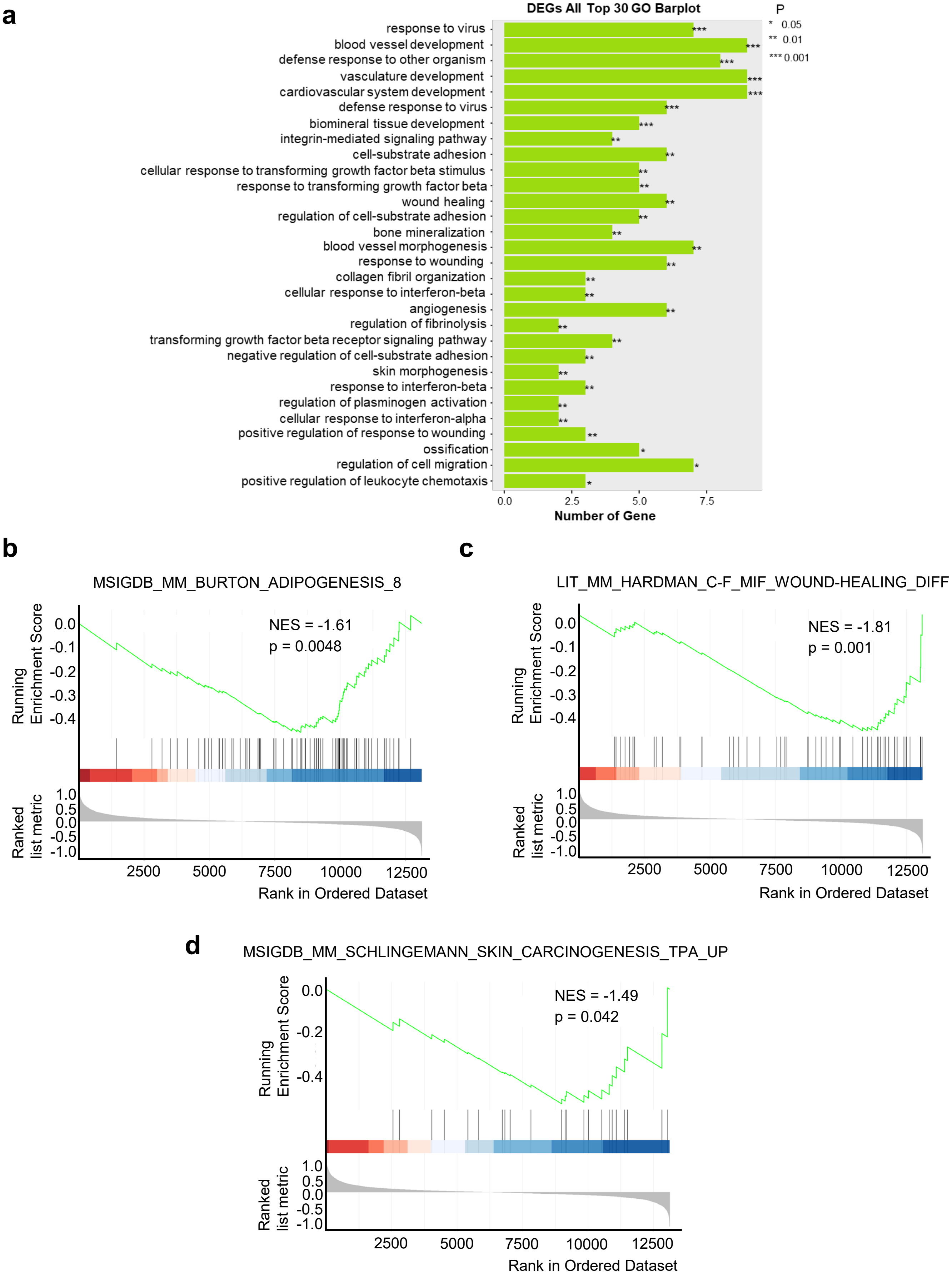
a
DEGs All Top 30 GO Barplot
P
* 0.05
** 0.01
*** 0.001
response to virus
blood vessel development
defense response to other organism
vasculature development
cardiovascular system development
defense response to virus
biomineral tissue development
integrin-mediated signaling pathway
cell-substrate adhesion
cellular response to transforming growth factor beta stimulus
response to transforming growth factor beta
wound healing
regulation of cell-substrate adhesion
bone mineralization
blood vessel morphogenesis
response to wounding
collagen fibril organization
cellular response to interferon-beta
angiogenesis
regulation of fibrinolysis
transforming growth factor beta receptor signaling pathway
negative regulation of cell-substrate adhesion
skin morphogenesis
response to interferon-beta
regulation of plasminogen activation
cellular response to interferon-alpha
positive regulation of response to wounding
ossification
regulation of cell migration
positive regulation of leukocyte chemotaxis
0.0 2.5 5.0 7.5
Number of Gene
b
MSIGDB_MM_BURTON_ADIPOGENESIS_8
NES = -1.61
p = 0.0048
Running Enrichment Score
Ranked list metric
Rank in Ordered Dataset
c
LIT_MM_HARDMAN_C-F_MIF_WOUND-HEALING_DIFF
NES = -1.81
p = 0.001
Running Enrichment Score
Ranked list metric
Rank in Ordered Dataset
d
MSIGDB_MM_SCHLINGEMANN_SKIN_CARCINOGENESIS_TPA_UP
NES = -1.49
p = 0.042
Running Enrichment Score
Ranked list metric
Rank in Ordered Dataset

Figure S5. Differentially expressed genes in BTG3-KO CM-differentiated adipocytes are preferentially associated with cell migration and skin carcinogenesis. **(a)** GO analysis following RNA-seq reveals significant association of DEGs in BTG3-KO CM-differentiated adipocytes with immune response, cell-substrate adhesion, cell migration, and angiogenesis. RNA was prepared from adipocytes differentiated in CM from parental or BTG3-KO HaCaT and subjected to RNA-seq analysis. **(b-d)** GSEA showing that the above DEGs are enriched in processes related to adipogenesis (b), wound healing (c), and TPA-induced skin carcinogenesis (d).

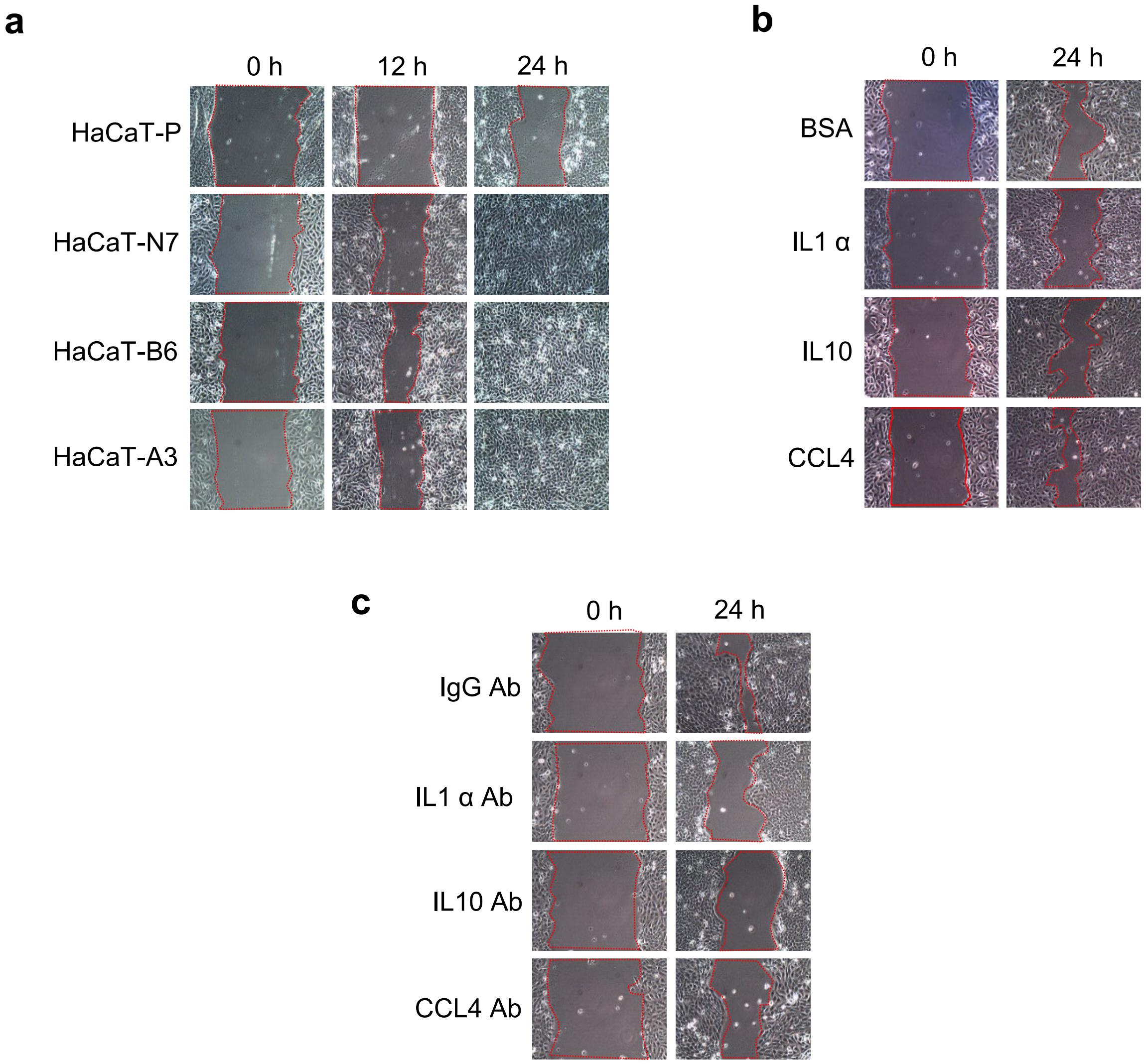

Fig. S6. BTG3 KO in keratinocytes promotes cell migration. **(a)** Cell migration was enhanced by the loss of BTG3 as revealed by wound healing assay. Images correspond to the results shown in Fig. S3f. **(b, c)** IL1 , IL10, and CCL4 promote parental HaCaT cell migration, assessed with wound healing assay. Images are in support of results shown in Fig. S3h (b) and Fig. S3i (c).

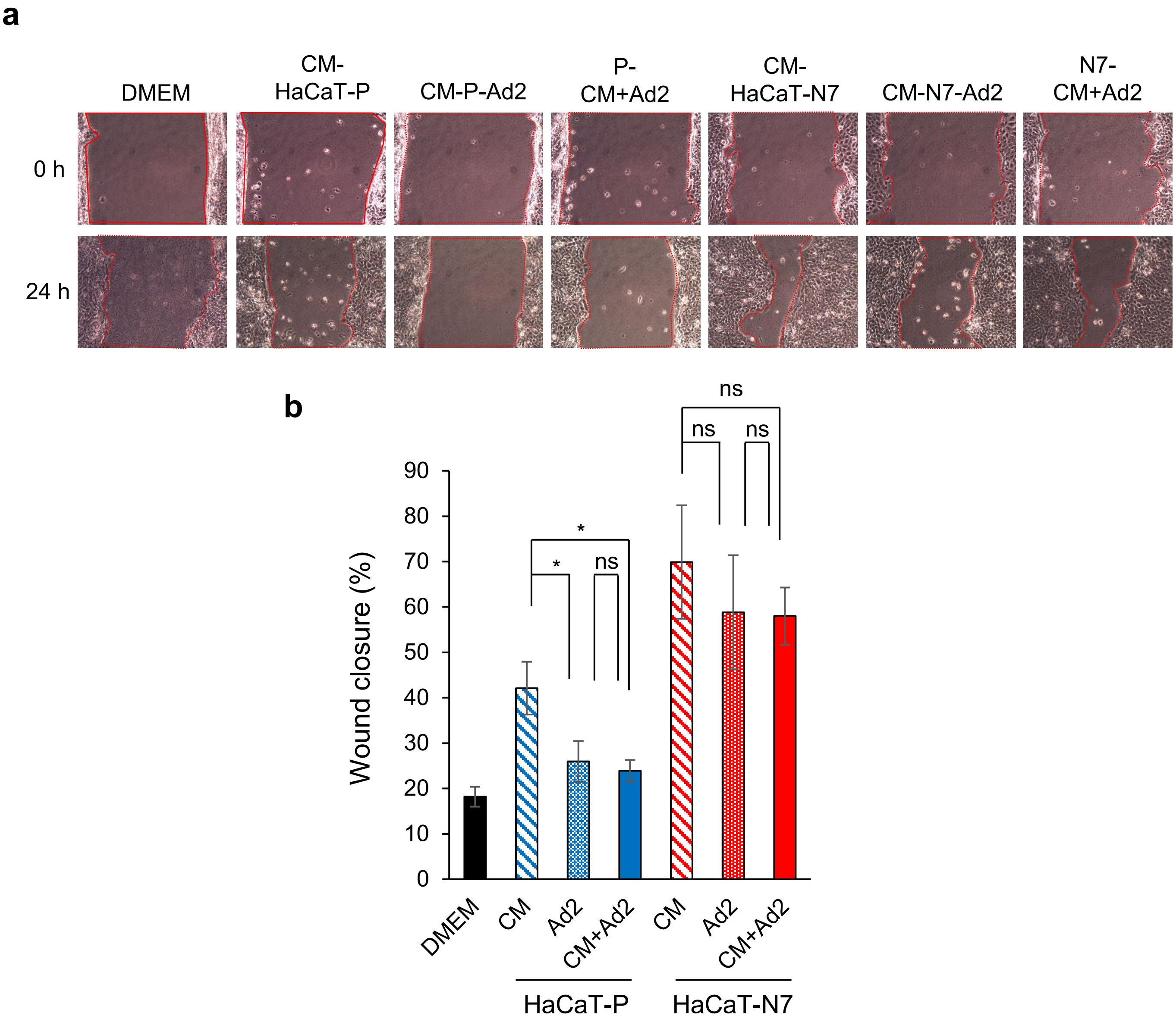

Fig. S7. Migration of HaCaT cells is promoted by parental and BTG3-KO CM, and such enhancement is significantly dampened by the addition of P-CM-Ad2 but not by BTG3-KO-Ad2. **(a)** Wound healing assays in DMEM (control) or the indicated CM, alone or in combination. **(b)** Quantification of the results shown in (a).

a

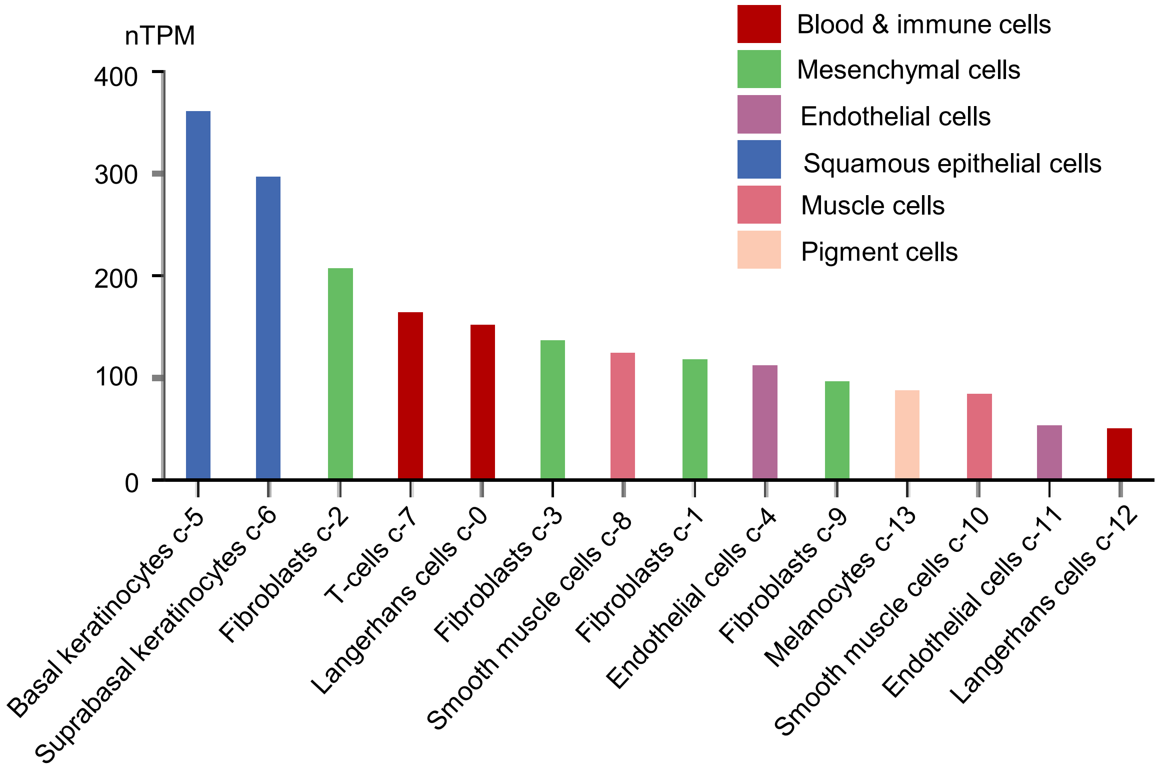

Figure S8. BTG3 is most abundantly expressed in basal keratinocytes compared with other cell types in the skin. Data were compiled from the Human Protein Atlas platform (https://www.proteinatlas.org/).

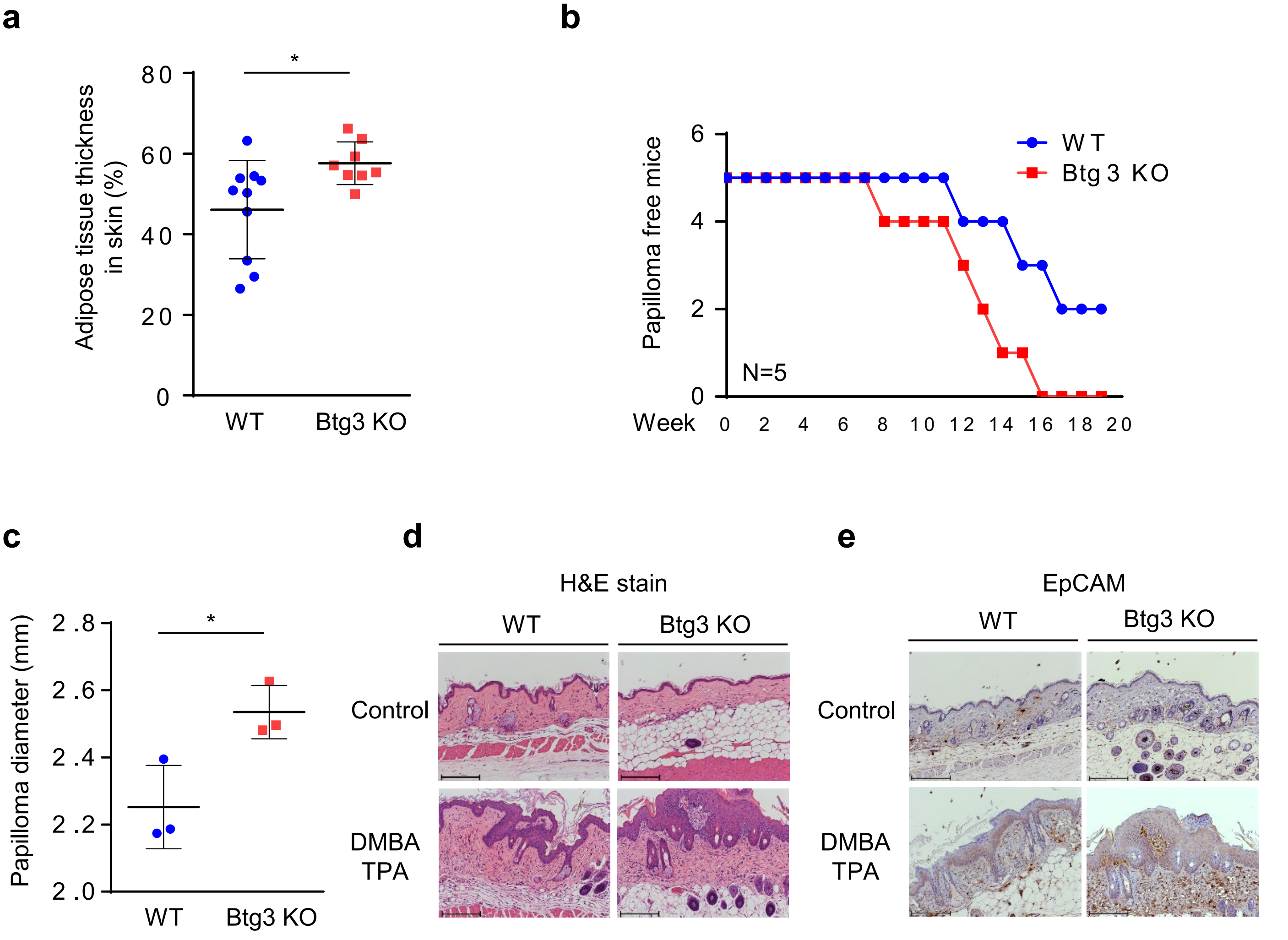

Figure S9. DMBA/TPA two-stage skin carcinogenesis in aged mice. **(a)** Thicker hypodermal adipose tissue in Btg3-/- compared with WT mice in the aged group (18 month-old). **(b)** Papillomas developed significantly earlier in Btg3-/- than in WT mice in the aged group. Mice of 18 month-old were treated with DMBA followed by TPA as in Fig. 5b. **(c)** Papillomas were larger in size in Btg3-/- mice. **(d)** Hematoxylin and eosin staining of control and papilloma-bearing skin from WT and Btg3-/- mice. **(e)** IHC staining of control and papilloma-bearing skin sections with the anti-EpCAM antibody.

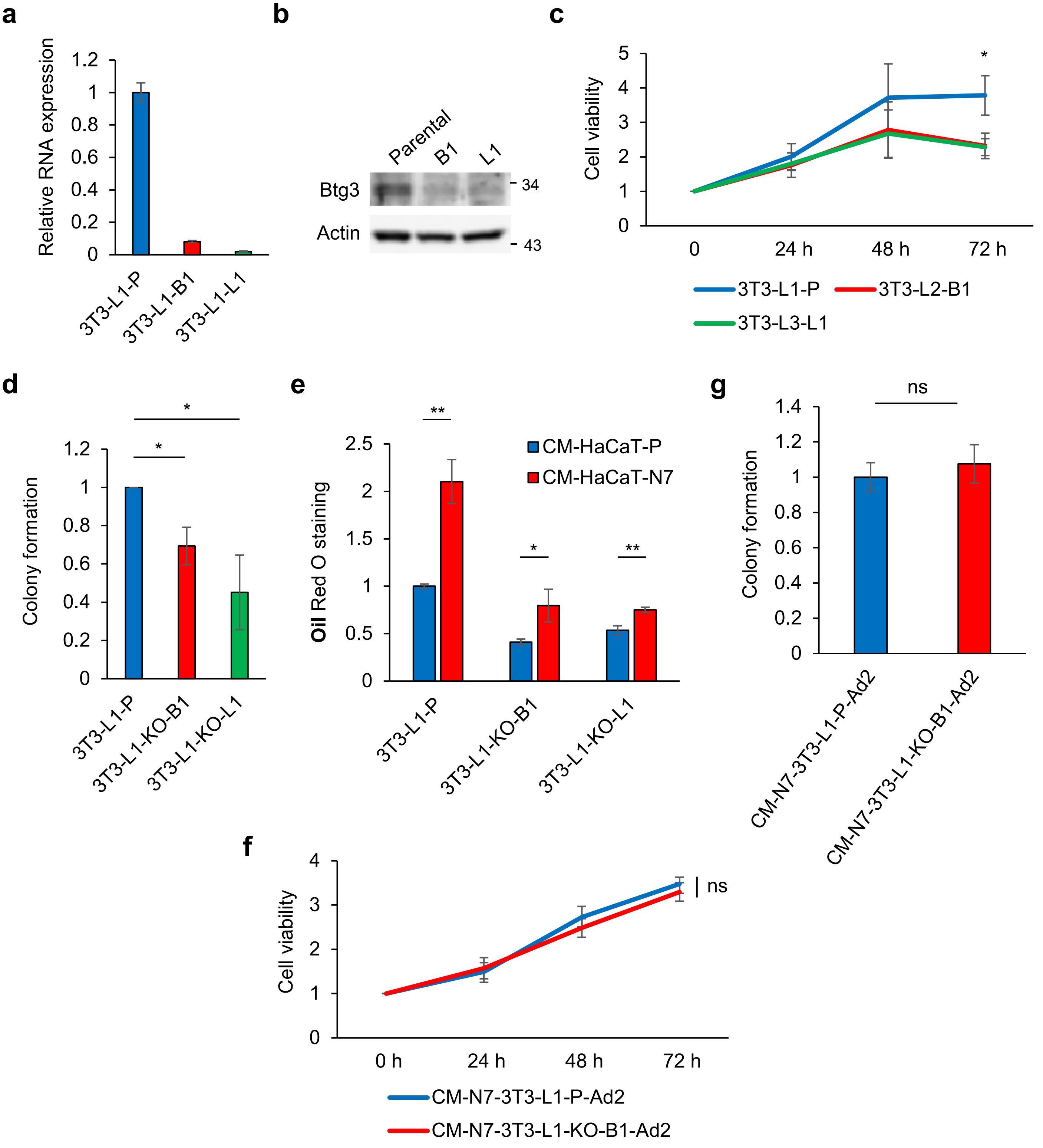
a
Relative RNA expression
1.2
1
0.8
0.6
0.4
0.2
0
3T3-L1-P
3T3-L1-B1
3T3-L1-L1
b
Parental
B1
L1
Btg3
Actin
34
43
c
Cell viability
5
4
3
2
1
0
*
0
24 h
48 h
72 h
3T3-L1-P
3T3-L2-B1
3T3-L3-L1
d
Colony formation
*
*
3T3-L1-P
3T3-L1-KO-B1
3T3-L1-KO-L1
e
Oil Red O staining
**
*
**
CM-HaCaT-P
CM-HaCaT-N7
3T3-L1-P
3T3-L1-KO-B1
3T3-L1-KO-L1
g
ns
Colony formation
CM-N7-3T3-L1-P-Ad2
CM-N7-3T3-L1-KO-B1-Ad2
f
Cell viability
ns
0 h
24 h
48 h
72 h
CM-N7-3T3-L1-P-Ad2
CM-N7-3T3-L1-KO-B1-Ad2

Fig. S10. BTG3 KO in 3T3-L1 causes reduced cell proliferation without affecting its paracrine effect on HaCaT cells. **(a)** Reduced expression of Btg3 in two of the CRISPR/Cas9 KO 3T3-L1 lines B1 and L1, revealed by RT-qPCR. **(b)** Western blot analysis showing the reduced Btg3 levels in B1 and L1 cells. **(c, d)** Btg3-KO-L1 and -B1 cells exhibited reduced cell viability (c) and colony formation (d) compared to the parental 3T3-L1 cells. **(e)** Adipocyte differentiation of B1 and L1 cells can still be promoted by BTG3-KO HaCaT CM. **(f, g)** Ad2 collected from the differentiation assay using Btg3 KO 3T3-L1 could support parental HaCaT proliferation (f) and colony formation (g) similar to that of parental 3T3-L1.

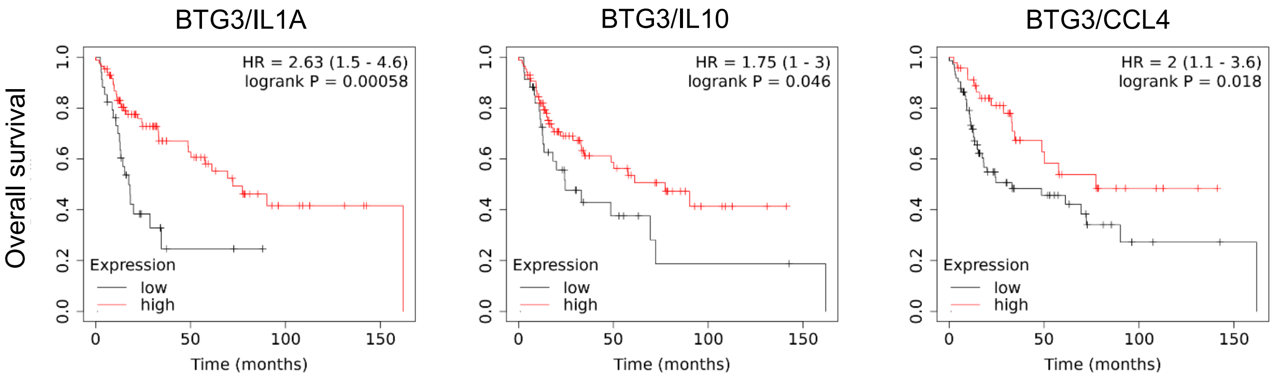

Number at risk

low	34	2	0	0
high	29	85	7	1

Number at risk

low	34	7	2	1
high	85	24	5	0

Number at risk

low	73	17	3	1
high	46	14	4	0

Figure S11. Low expression ratios of BTG3 over IL1A, IL10, or CCL4 correlate with poor patient survival in high-grade HNSCC. Overall survival was analyzed using the Kaplan-Meier Plotter (http://kmplot.com/analysis/) platform.
