# Supplementary figures and images for: A keratinocyte-adipocyte signaling loop is reprogrammed by loss of BTG3 to augment skin carcinogenesis
Source: Cell Death Differ. 2024 May 7;31(8):970–82. doi: 10.1038/s41418-024-01304-7 (PMC11303697; doi:10.1038/s41418-024-01304-7)

Figure 1d

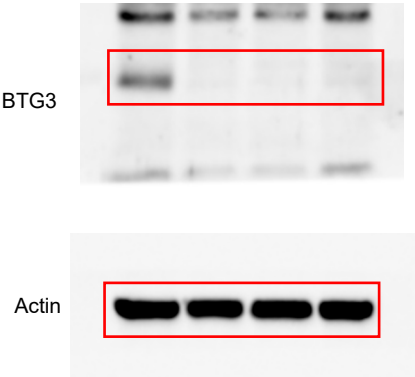

Figure 3c

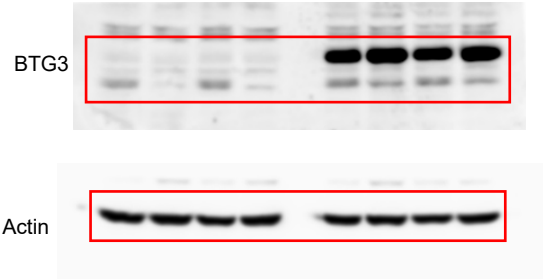

Figure 3d

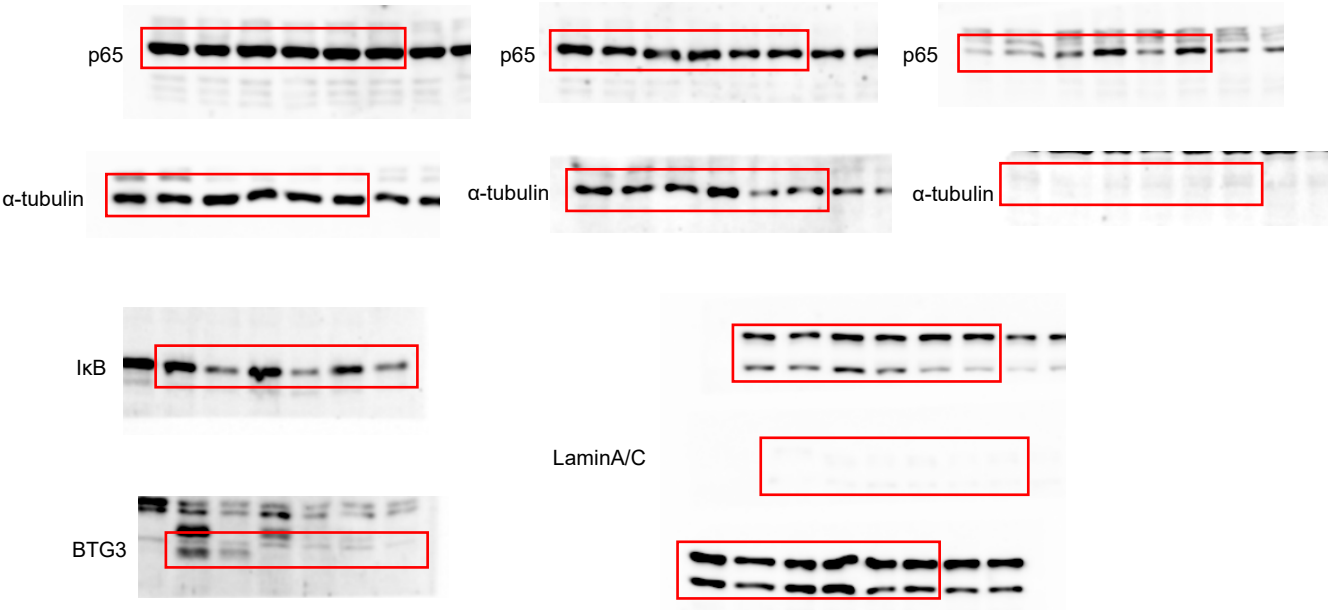

**Figure 3e**

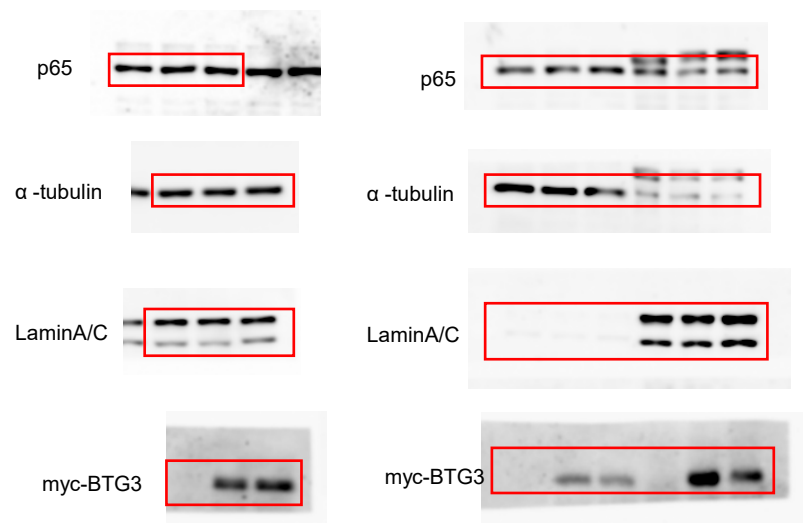

**Figure 3f**

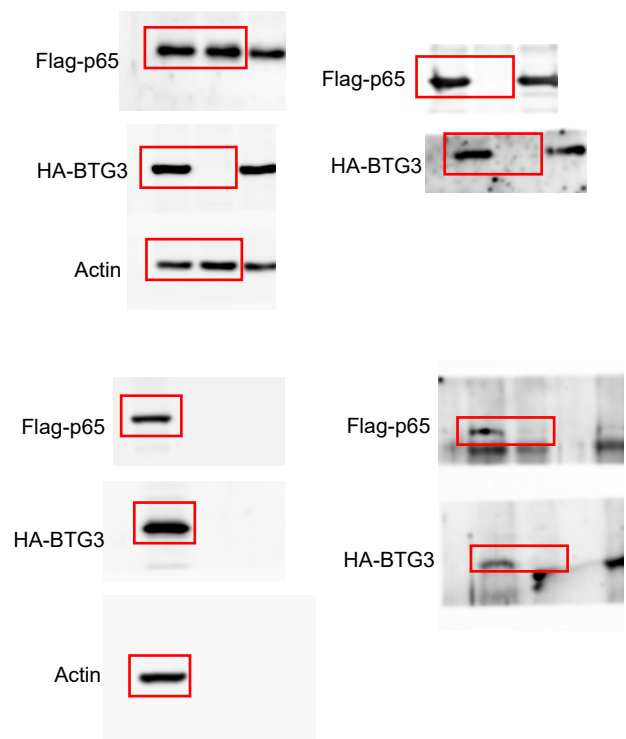

**Figure 3g**

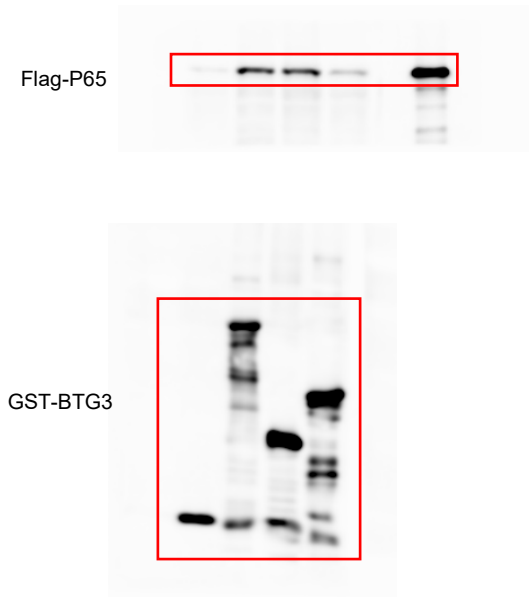

**Figure 3h**

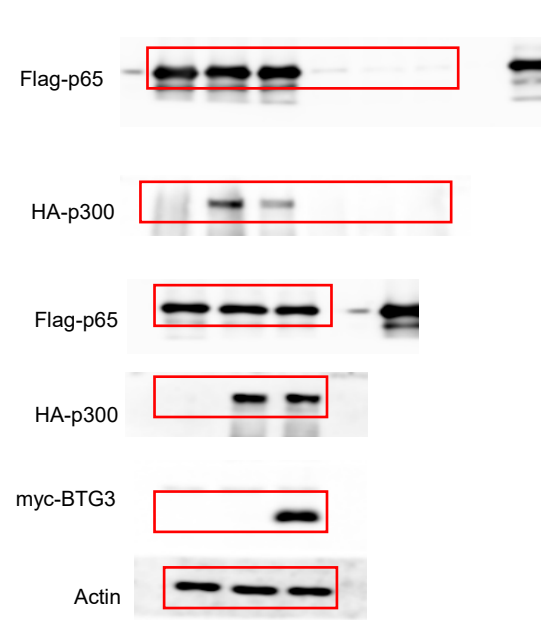

**Figure S3d**

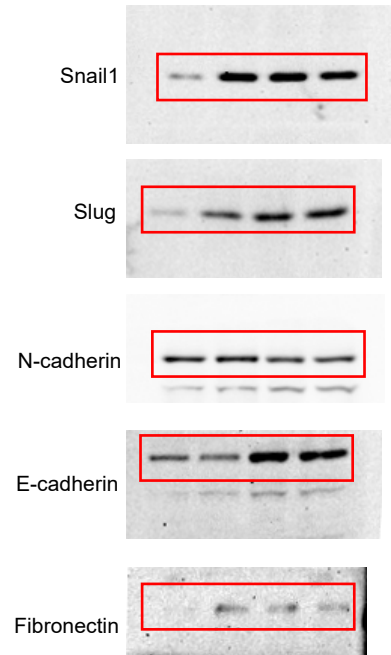

**Figure S10b**

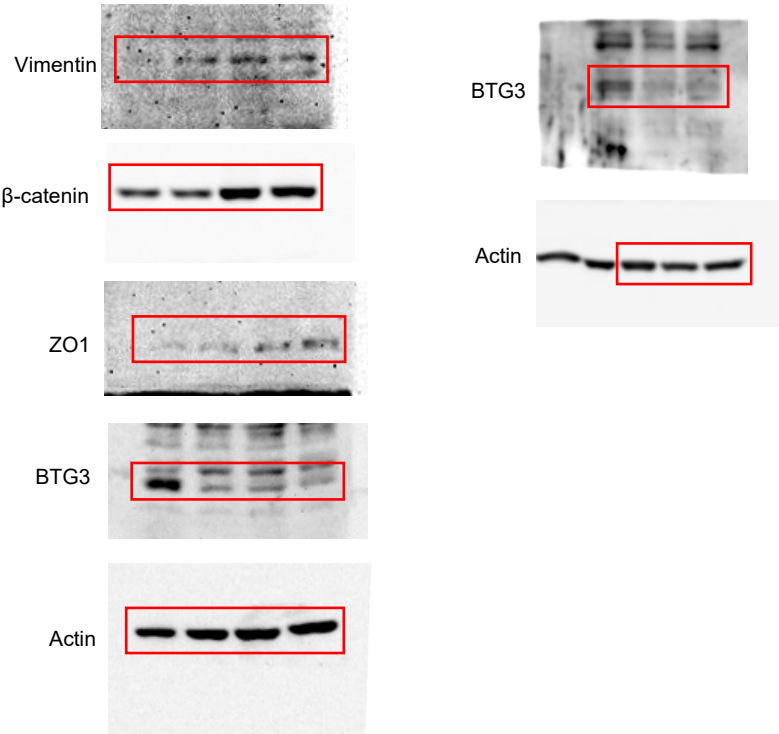

Supplement: Supplementary file 4 — Original data files [file 41418_2024_1304_MOESM4_ESM.pdf]
